# The deubiquitinase USP21 maintains the stemness of mouse embryonic stem cells via stabilization of Nanog

**DOI:** 10.1038/ncomms13594

**Published:** 2016-11-25

**Authors:** Jiali Jin, Jian Liu, Cong Chen, Zhenping Liu, Cong Jiang, Hongshang Chu, Weijuan Pan, Xinbo Wang, Lingqiang Zhang, Bin Li, Cizhong Jiang, Xin Ge, Xin Xie, Ping Wang

**Affiliations:** 1 Shanghai Key Laboratory of Regulatory Biology, Institute of Biomedical Sciences and School of Life Sciences, East China Normal University, 500 Dongchuan Road, Shanghai 200241, China; 2 Chinese Academy of Sciences Key Laboratory of Receptor Research, National Center for Drug Screening, Shanghai Institute of Materia Medica, Chinese Academy of Sciences, Shanghai 201203, China; 3 University of Chinese Academy of Sciences No. 19A Yuquan Road, Beijing 100049, China; 4 Department of Central Laboratory, Shanghai Tenth People's Hospital of Tongji University, School of Life Science and Technology, Tongji University, Shanghai 200072, China; 5 State Key Laboratory of Proteomics, Beijing Proteome Research Center, Beijing Institute of Radiation Medicine, Collaborative Innovation Center for Cancer Medicine, Beijing 100850, China; 6 Key Laboratory of Molecular Virology and Immunology, Unit of Molecular Immunology, Institute Pasteur of Shanghai, Shanghai Institutes for Biological Sciences, Chinese Academy of Sciences, Shanghai 200031, China; 7 Department of Clinical Medicine, Shanghai Tenth People's Hospital of Tongji University, Tongji University, Shanghai 200072, China

## Abstract

Nanog is a master pluripotency factor of embryonic stem cells (ESCs). Stable expression of Nanog is essential to maintain the stemness of ESCs. However, Nanog is a short-lived protein and quickly degraded by the ubiquitin-dependent proteasome system. Here we report that the deubiquitinase USP21 interacts with, deubiquitinates and stabilizes Nanog, and therefore maintains the protein level of Nanog in mouse ESCs (mESCs). Loss of USP21 results in Nanog degradation, mESCs differentiation and reduces somatic cell reprogramming efficiency. USP21 is a transcriptional target of the LIF/STAT3 pathway and is downregulated upon differentiation. Moreover, differentiation cues promote ERK-mediated phosphorylation and dissociation of USP21 from Nanog, thus leading to Nanog degradation. In addition, USP21 is recruited to gene promoters by Nanog to deubiquitinate histone H2A at K119 and thus facilitates Nanog-mediated gene expression. Together, our findings provide a regulatory mechanism by which extrinsic signals regulate mESC fate via deubiquitinating Nanog.

## Abstract (web summary)

Nanog regulates embryonic stem cell (ESC) pluripotency but what controls Nanog protein stability is unclear. Here, the authors show that in mouse ESCs, Nanog protein is ubiquitinated and stabilized by the deubiquitinase USP21, which in turn is regulated by extrinsic signals, STAT3 and ERK.

## Graphical abstract

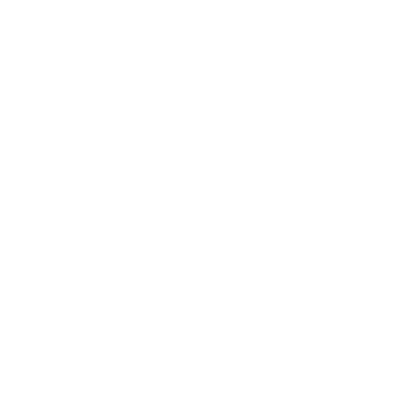

The pluripotency of embryonic stem cells (ESCs) is regulated by a network of transcription factors (TFs), including Oct4, Sox2 and Nanog^1^. Among them, Nanog plays an essential role in the transcriptional network of pluripotency and early embryonic development^2^,^3^, controlling the epiblast versus primitive endoderm decision in the blastocyst^4^. Nanog-depleted blastocysts fail to generate epiblasts and produce only parietal endoderm-like cells^3^, whereas the ectopic expression of Nanog is sufficient to induce leukaemia inhibitory factor (LIF)-independent self-renewal of cultured mouse ESCs (mESCs)^2^. Downregulation of Nanog in mESCs results in differentiation into a broader repertoire of cell lineages, with a primary contribution to the trophectoderm and primitive endoderm^5^. Due to its essential roles in pluripotency, Nanog is among the four factors (Oct4, Sox2, Nanog and Lin28) that were initially used to reprogram human somatic cells into pluripotent stem cells^6^.

The amounts of the pluripotency factors in ESCs are precisely controlled to maintain ESCs self-renewal^5^,^7^,^8^. A number of TFs have been reported to activate and/or repress Nanog expression in mESCs, with Oct4 and Sox2 representing the major regulators^9^. Fine-tuning of Nanog expression is achieved via several signal transduction cascades. For instance, Nanog expression is promoted by LIF through two parallel pathways: the JAK/STAT3 pathway, which activates Klf4, and the PI3K/AKT pathway, which activates Tbx3 (ref. 10). A STAT3-binding site has been identified in an enhancer region upstream of the Nanog promoter^11^. Other signalling pathways, such as FGF/MEK^12^, GSK3β^13^ and TGFβ^14^, also participate in the regulation of Nanog expression. Interestingly, Nanog expression is primarily monoallelic in mESCs cultured in standard LIF/serum conditions. However, culturing mESCs in 2i (MEK inhibitor and GSK3 inhibitor)/LIF conditions significantly increases the level of biallelic Nanog expression, indicating that Nanog is a marker of ground-state pluripotency^15^,^16^.

In addition to the regulation of their expression, the degradation of these pluripotency factors is also tightly controlled by the ubiquitin–proteasome system (UPS). The UPS is one of the key systems that regulate cellular protein levels under various conditions. Protein ubiquitination is a reversible and balanced process catalysed by Ub-activating enzyme (E1), Ub-conjugating enzyme (E2), Ub-protein ligase (E3) and deubiquitinases (DUBs). Several studies have investigated the mechanism by which the UPS controls the protein levels of key pluripotency factors. As a key pluripotency factor, Nanog is a short-lived protein that is rapidly degraded by E3 ligase FBXW8-mediated ubiquitination^17^. However, the DUBs that regulate Nanog stability are unknown.

In this study, we sought to identify DUBs that specifically regulate Nanog stability. We screened 46 DUBs and identified USP21 as an efficient deubiquitinating enzyme that governs Nanog stability in ESCs. Our study thus reveals a dynamic regulatory mechanism underlying Nanog stability and transcriptional activity through external signals.

## Results

### The DUB USP21 regulates Nanog stability

**Figure 1:**
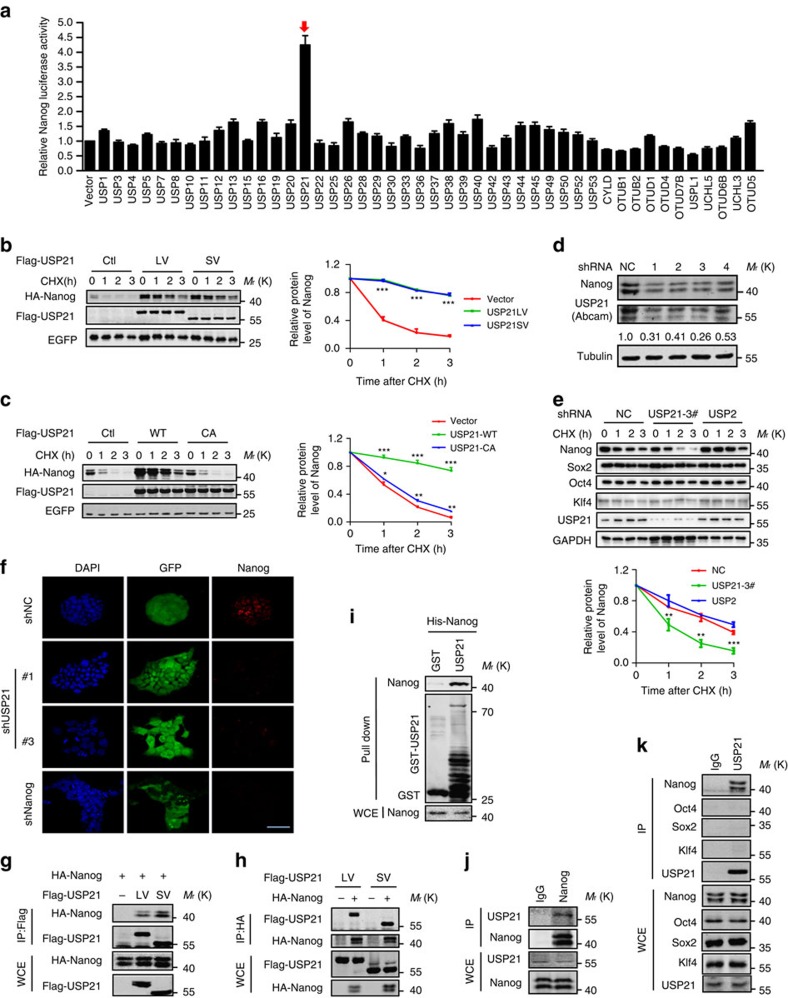
USP21 directly stabilizes and interacts with Nanog. (**a**) Screening for the deubiquitinating enzymes of Nanog. HEK293T expressing the indicated proteins, the firefly luciferase activity was normalized to the renilla luciferase activity and then normalized to vector control. (**b**) HA-Nanog was co-expressed in HEK293T with the vector, Flag-USP21-LV or Flag-USP21-SV. After treating the cells with cycloheximide (CHX, 10 μg ml^−1^) for indicated time intervals, associated protein levels were analysed by western blotting (left panel). Statistical analysis of Nanog was presented on the right. Data are means±s.d. (*n*=3), ***P<0.001 versus vector (Student's *t*-test). (**c**) Flag-Nanog was co-expressed in HEK293T with vector, HA-USP21 WT or HA-USP21-C221A. After treating cells with cycloheximide (CHX, 10 μg ml^−1^) for indicated time intervals, the protein levels were analysed by western blotting (left panel). Statistical analysis of Nanog was presented on the right. Data are means±s.d. (*n*=3), *P<0.05, **P<0.01, ***P<0.0001 versus vector (Student's *t*-test). (**d**) The knockdown efficiency of USP21 in E14 cells. (**e**) E14 cells were transfected with control shRNA, *Usp21* shRNA3 or *Usp2* shRNA and then treatment with CHX (10 μg ml^−1^). The endogenous protein levels were analysed by western blotting (top panel, the anti-USP21 antibody is from Abgent), and statistical analysis of Nanog was presented on the bottom. Data are means±s.d. (*n*=3), **P<0.01, ***P<0.001 versus shNC (Student's *t*-test). (**f**) E14 cells were infected with virus carrying GFP together with control shRNA or shRNA targeting USP21 or Nanog, and Nanog was detected with immunocytochemical staining on day 3. Scale bar, 50 μm. (**g**,**h**) Flag-USP21 and HA-Nanog were co-expressed in HEK293T cells. USP21 and Nanog were immunoprecipitated with anti-Flag (**g**) or HA (**h**) antibody, respectively, and the associated Nanog and USP21 were analysed by western blotting using either HA or Flag antibody. (**i**) GST pull-down assays indicated that USP21 interacts with Nanog directly. (**j**) Endogenous Nanog was immunoprecipitated with an antibody against Nanog from E14 cells, and the associated USP21 was detected by an anti-USP21 antibody obtained from Abgent. (**k**) Endogenous USP21 was immunoprecipitated with an antibody against USP21 (Abgent) from E14 cells. Nanog, Sox2, Oct4 and Klf4 were detected by western blotting.

To search for DUBs that could stabilize Nanog, we fused firefly luciferase to the C terminus of Nanog and used this fusion protein (Nanog-Luc) as a reporter of Nanog stability (Supplementary Fig. 1a). As the fused luciferase is degraded together with the Nanog, the degradation of Nanog can be easily quantified by measuring the luciferase activity. Forty-six mammalian DUBs were screened with this reporter. We found that co-expression with USP21, but not the other DUBs, significantly increased the luciferase activity of Nanog-Luc (Fig. 1a).

Two USP21 isoforms (USP21-LV and USP21-SV) with different subcellular locations have been reported (Supplementary Fig. 1b)^18^. Co-expression of either USP21 isoform significantly prolonged the half-life of Nanog (Fig. 1b). In contrast, USP21 had almost no effect on the half-life of Oct4, Klf4 and Sox2 protein (Supplementary Fig. 1c). The stabilization of Nanog by USP21 was dependent on its deubiquitinating enzyme activity because the catalytic-inactive mutant C221A (CA-USP21) neither stabilized Nanog (Fig. 1c) nor increased the Nanog transcriptional activity measured by a luciferase reporter assay (Supplementary Fig. 1d). Depletion of endogenous USP21 by short hairpin RNA (shRNA) in mESC E14 cells reduced both the protein level (Fig. 1d) and half-life of Nanog, but not the half-life of other pluripotent factors such as Sox2, Oct4 and Klf4 (Fig. 1e). It is interesting to note that knockdown of USP21 led to reduced protein levels of Nanog, Sox2, Oct4 and Klf4, indicating a loss of pluripotency. To ensure the starting protein levels of these factors were similar in the protein half-life assay, we increased the loading amount of the USP21 knockdown group by twofold (which is why you see an increase in the GAPDH level). The half-life of Nanog was also shortened in USP21 knockout (USP21-KO) mouse embryonic fibroblasts (MEFs; Supplementary Fig. 1e, see below). In addition of the loss of Nanog protein, knockdown USP21 also led to the loss of the tight compact morphology of mESC (Fig. 1f).

To examine whether Nanog is specifically regulated by USP21, we examined the regulation of Nanog by USP2, the most closely related DUB to USP21. Our data showed the overexpression of USP2 slightly prolonged the half-life of Nanog (Supplementary Fig. 1f). However, knockdown USP2 in E14 cells had little effect on the half-life of Nanog (Fig. 1e), indicating a less significant role of USP2 on Nanog stability in native condition. Knockdown of USP2 and USP21 was monitored by PCR with reverse transcription (Supplementary Fig. 1g). Together, these data indicate that USP21, but not USP2, is a specific DUB that regulates Nanog stability.

### USP21 directly interacts with and deubiquitinates Nanog

Since USP21 is a DUB, we examined whether USP21 could directly interact with and deubiquitinate Nanog. Both USP21 isoforms were found to bind to Nanog and vice versa when examined by co-immunoprecipitation (co-IP) assay (Fig. 1g,h). In contrast, the binding between Nanog and USP2 was much weaker than that between Nanog and USP21 (Supplementary Fig. 1h). The glutathione *S*-transferase (GST) pull-down assay using purified Nanog and USP21 proteins demonstrated that USP21 directly interacted with Nanog (Fig. 1i). In E14 cells, the endogenous USP21 was also found to interact with endogenous Nanog (Fig. 1j), but not other stem cell factors (Oct4, Sox2 and Klf4; Fig. 1k). The results were confirmed using an overexpression system in HEK293T cells (Supplementary Fig. 1i). Together, these data indicate that USP21 is a direct binding partner for Nanog.

To determine the region(s) of USP21 and Nanog responsible for their interaction, several deletion mutants of USP21 and Nanog were generated, and their interactions were examined using co-IP assays. The C-terminal UCH (catalytic domain), but not the N-terminal domain of USP21 was found to be responsible for its interaction with Nanog (Supplementary Fig. 1j). Mouse Nanog contains serine-rich N-terminal (N), NK-2-type homeobox (H) and C-terminal (C1WC2) domains. We found that the C-terminal domain of Nanog was required for its binding to USP21 (Supplementary Fig. 1k).

**Figure 2:**
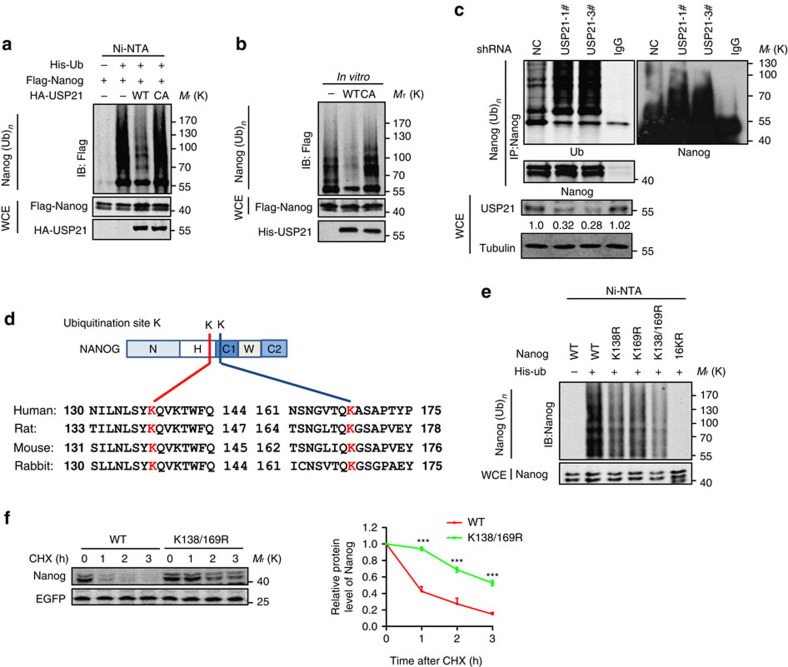
USP21 deubiquitinates Nanog. (**a**) Flag-Nanog and His-ubiquitin were co-expressed with USP21 WT or USP21-C221A in HEK293T cells. After MG132 (10 μM) treatment for 6 h, the ubiquitinated proteins were pulled down under denaturing conditions using Ni-NTA agarose beads and the ubiquitination of Nanog was detected by western blotting using an anti-Flag antibody. (**b**) His-USP21 protein was purified from *E. coli*, Flag-Nanog was purified by immunoprecipitated from HEK293T cells were transfected with Flag-Nanog, followed by immunoprecipitation using an anti-Flag antibody and then subjected to *in vitro* deubiquitination. (**c**) E14 cells stably expressing control or USP21 shRNA1 and USP21 shRNA3 were treated with MG132 (10 μM) for 6 h. Nanog was immunoprecipitated with an anti-Nanog antibody and the ubiquitination of Nanog was examined by western blotting using an anti-ubiquitin antibody. USP21 was detected by an anti-USP21 antibody from Abgent. (**d**) Alignment of the potential ubiquitin site in Nanog from different species. (**e**) WT or lysine-mutated Nanog and His-ubiquitin were co-expressed in HEK293T cells. After treatment with MG132 (10 μM) for 6 h, the ubiquitinated proteins were pulled down under denaturing conditions using Ni-NTA agarose beads and the ubiquitination of Nanog was detected by western blotting using the anti-Nanog antibody. (**f**) WT or K138/169R Nanog was expressed or co-expressed with Flag-USP21 in HEK293T cells. After treatment with CHX (10 μg ml^−1^), the protein level of Nanog was analysed by western blotting (left panel), and statistical analysis was presented on the right. Data are means±s.d. (*n*=3), ***P<0.001 versus WT (Student's *t*-test).

Next, we examined whether USP21 could deubiquitinate Nanog. Our data showed that co-expression of the wild type (WT)-USP21, but not the CA-USP21, almost completely removed the ubiquitin chains from Nanog (Fig. 2a; Supplementary Fig. 2a). In addition, the deubiquitination ability of USP21 was stronger than USP2 (Supplementary Fig. 2a). To examine whether USP21 deubiquitinated Nanog directly, both WT-USP21 and CA-USP21 were purified and their ability to deubiquitinate Nanog was tested in an *in vitro* deubiquitination assay. We found that WT-USP21, but not CA-USP21, efficiently deubiquitinated Nanog (Fig. 2b), indicating that USP21 was a direct DUB for Nanog. Moreover, depletion of USP21 by shRNAs in mESCs significantly increased the ubiquitination of endogenous Nanog (Fig. 2c), indicating that endogenous Nanog was also a target of USP21.

Mouse Nanog contains 16 lysines. Mutation of all of these lysine residues (16KR) completely abolished Nanog ubiquitination (Fig. 2e), indicating that Nanog was ubiquitinated at lysine residues. Nanog has been reported to be ubiquitinated at K138 and K169 residues (http://www.phosphosite.org/proteinAction.do?id=15240190&showAllSites=true), which are conserved among various species including humans, rats and rabbits (Fig. 2d). Our data confirmed that the simultaneous replacement of K138 and K169 with Arg (K138/K169R) markedly reduced Nanog ubiquitination (Fig. 2e). A Nanog double mutant (K138/K169R) also showed a longer half-life than WT Nanog (Fig. 2f). The K to R mutations did not affect the interaction between USP21 and Nanog (Supplementary Fig. 2b), suggesting that binding of USP21 was independent of Nanog ubiquitination. Consistently with its stability, the Nanog K138/K169R mutant showed slightly stronger transcriptional activity than WT Nanog (Supplementary Fig. 2c). Together, these data indicate that USP21 is a bona fide DUB of Nanog.

### USP21 regulates mESC function

**Figure 3:**
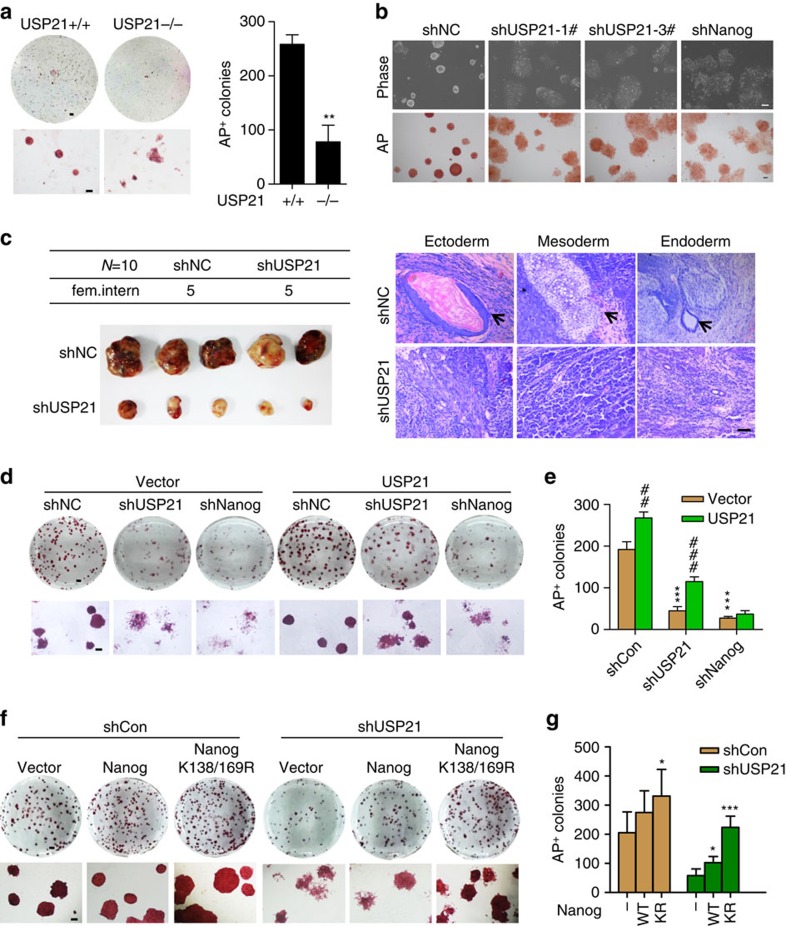
USP21 is required for mESC self-renewal. (**a**) *Usp21*^Floxp/Floxp^ ESCs were derived from *Usp21*^Floxp/Floxp^ mice and *USP21* was depleted by infection with lentivirus carrying Cre recombinase. *USP21*^+/+^ and *USP21*^−/−^ ESCs were stained with AP. Scale bar, 1 mm (for upper panel); 100 μm (for lower panel). Experiment was repeated three times. Shown are average values of triplicated results with means±s.d. **P<0.01 versus USP21^+/+^ (Student's *t*-test). (**b**) Morphology and AP staining of E14 cells infected with virus carrying control or specific shRNAs targeting USP21 or Nanog for 4 days. Scale bar, 100 μm. (**c**) Teratoma formation from E14 cells infected with control or USP21 shRNA. Scale bar, 100 μm. Right: haematoxylin and eosin staining showing tissues derived from three germ layers. Left: size of the teratoma. (**d**,**e**) AP staining (left) and statistical analysis (right) of E14 cells infected with shRNA targeting 3′UTR of USP21 or Nanog for 2 days and then rescued with shRNA-resistant USP21 for another 2 days. Scale bar, 1 mm (for upper panel); 50 μm (for lower panel). Data are means±s.d. (*n*=3). ***P<0.001 versus shCon (two-way analysis of variance (ANOVA) test); ^##^*P*<0.01, ^###^*P*<0.001 versus vector (two-way ANOVA test). (**f**,**g**) AP staining (left) and statistical analysis (right) of E14 cells infected with USP21 shRNA for 2 days and then rescued with WT or K138/169R Nanog for another 2 days. Scale bar, 1 mm (for upper panel); 50 μm (for lower panel). Data are means±s.d. (*n*=3), *P<0.05, **P<0.01 (two-way ANOVA test), ***P<0.001 versus vector (two-way ANOVA test).

Nanog is essential for the maintenance of mESC self-renewal and pluripotency^19^. Thus, we examined whether USP21 plays a role in the self-renewal of mESCs. We derived *Usp21*^Loxp/Loxp^ ESCs by intercrossing *Usp21*^Loxp/Loxp^ mice; *Usp21* was knocked out by infection with a lentivirus carrying Cre recombinase (Supplementary Fig. 3a,b). For mESCs, the colonies are typically tightly packed, bright, dome-shaped with smooth edge. In alkaline phosphatase (AP) staining, the pluripotent mESC colonies will be stained deeply in a reddish brown colour with smooth edge, while differentiating ESC colonies (much less compact compared with the pluripotent ones) will stain in less saturated colour or completely lose AP staining^20^. We found that deletion of USP21 caused the loss of mESC morphology and exhibited reduced AP staining (Fig. 3a). Moreover, USP21 knockdown with specific shRNAs in E14 cells also led to the loss mESC morphology and reduced AP staining (Fig. 3b; Supplementary Fig. 3c). Consistently, USP21 knockdown significantly reduced the expression of the pluripotency genes including *Nanog*, *Oct4* and *Rex1*, but increased the expression of lineage-specific genes, including the ectoderm markers *Fgf5* and *Nestin*, mesoderm marker *Desmin*, and endoderm markers *Gata4* and *Sox17*, in a pattern similar to Nanog depletion (Supplementary Fig. 3d), indicating that USP21 is required for the self-renewal of mESCs. Furthermore, *Usp21* depletion reduced the ability of mESCs to form teratomas (Fig. 3c). Conversely, overexpression of either USP21-LV or USP21-SV promoted mESC self-renewal under less optimal culture conditions. LIF withdrawal led to the significant differentiation of the control mESCs in 3 days. However, mESCs overexpressing USP21 were still able to maintain the typical mESC morphology and remain AP-positive in culture without LIF for 3 days (Supplementary Fig. 3e). Western blotting and PCR analysis also revealed the upregulation of pluripotency genes in mESCs overexpressing USP21 (Supplementary Fig. 3f). Although overexpression of USP21 promoted mESC self-renewal *in vitro*, it did not affect the developmental potential of these cells *in vivo*. Tissues derived from all three germ layers were found in teratomas formed with these USP21-overexpressing mESCs (Supplementary Fig. 3g).

Next, we investigated whether USP21 maintained mESC self-renewal by regulating Nanog. To this end, USP21 was knocked down with a specific shRNA targeting the 3′-untranslated region (UTR) of USP21; then, a shRNA-resistant USP21 was reintroduced. Reintroduction of USP21 efficiently rescued the mESC differentiation caused by USP21 depletion, but failed to rescue mESC differentiation caused by Nanog knockdown (Fig. 3d,e). Next, we examined whether the ubiquitination of Nanog contributed to ESC self-renewal. We found that the ubiquitination-resistant K138/K169R mutant had stronger ability to maintain the self-renewal of mESCs than WT Nanog. Moreover, the K138/169R mutant showed a stronger ability to rescue the effect of USP21 knockdown on mESC self-renewal (Fig. 3f,g). These data indicate that USP21 is required for the self-renewal of mESCs and Nanog is downstream of USP21.

Nanog is required to attain the ground state of authentic pluripotency in the final phase of somatic cell reprogramming^21^. Therefore, we examined whether USP21 was required for the generation of induced pluripotent stem cells (iPSCs) from MEFs with Oct4, Sox2 and Klf4. USP21-KO MEFs displayed a significantly lower reprogramming efficiency than WT MEFs, as indicated by the reduced number of AP-positive colonies on day 12 of induction (Supplementary Fig. 3h). On the other hand, overexpression of USP21-LV or USP21-SV in OG2 MEFs carrying an *Oct4*-GFP reporter significantly increased the number of GFP^+^ iPSC colonies induced by Oct4, Sox2, Klf4 and Nanog on day 16. In contrast, CA-USP21 had no effect (Supplementary Fig. 3i). Collectively, our data indicate that USP21 is a specific DUB for Nanog and plays important roles in mESC self-renewal and somatic cell reprogramming by regulating Nanog stability.

### USP21 is a transcriptional target of LIF/STAT3

**Figure 4:**
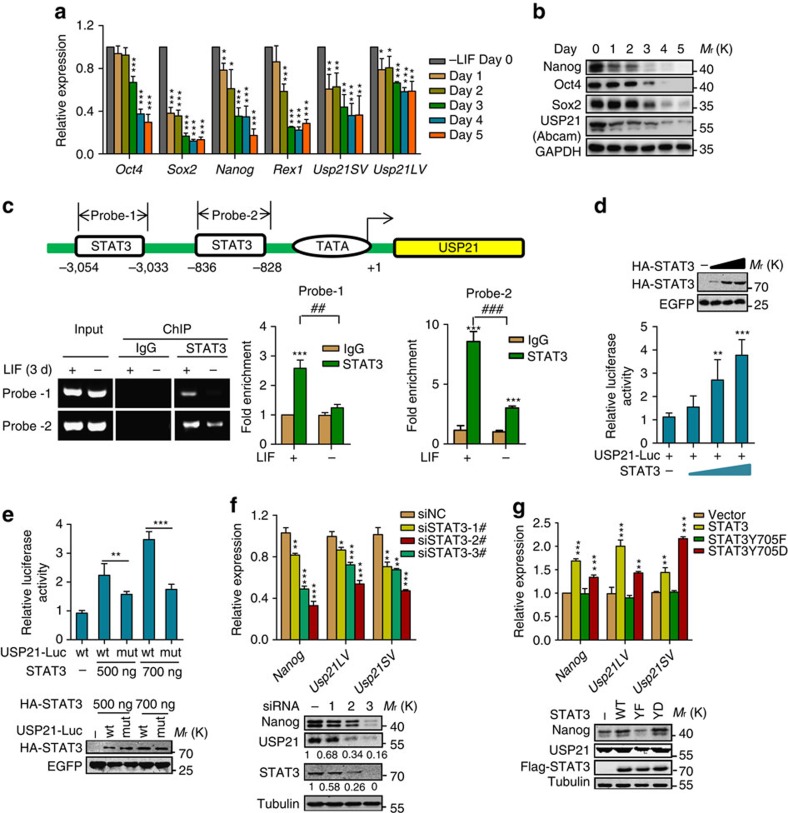
USP21 is a transcriptional target of LIF/STAT3. (**a**,**b**) Real-time PCR (**a**) and immunoblot (**b**) analyses of pluripotency markers and USP21 in E14 cells cultured in LIF-free condition for 5 days. Data are means±s.d. (*n*=3), *P<0.05, **P<0.01, ***P<0.001 versus day 0 (Student's *t*-test). The anti-USP21 antibody is from Abcam. (**c**) Upper: map of the USP21 promoter and the putative STAT3-binding sites. Bottom: ChIP–PCR analysis of E14 cultured with or without LIF for 3 days using anti-STAT3 antibody and PCR primers. IgG was used as a negative control. Enrichment of STAT3 on the USP21 promoter was calculated. Data are means±s.d. (*n*=3). ***P<0.001 versus IgG (two-way analysis of variance (ANOVA) test); ^##^*P*<0.01, ^###^*P*<0.001 versus LIF(+) condition (two-way ANOVA test). (**d**) HEK293T cells were transfected with STAT3 or vector control, plus the USP21 basic promoter-Luc reporter. Protein level (top) and luciferase activity of STAT3 were measured. Data are means±s.d. (*n*=3). **P<0.01, ***P<0.001 versus STAT3 (−) condition (Student's *t*-test). (**e**) HEK293T cells were transfected with STAT3 and USP21 basic promoter (WT or carrying mutations in the putative STAT3-binding site) Luc reporter. Luciferase activity (top) and protein level of STAT3 (bottom) were measured. Data are means±s.d. (*n*=3). **P<0.01, ***P<0.001 (Student's *t*-test). (**f**) Real-time PCR and immunoblot analysis of USP21 messenger RNA (mRNA) level and protein level after STAT3 knockdown for 3 days in E14 cells. Data are means±s.d. (*n*=3). *P<0.05, **P<0.01 (Student's *t*-test), ***P<0.001 versus siNC (Student's *t*-test). USP21 were detected by an anti-USP21 antibody from Abgent. (**g**) E14 cells transfected with WT or STAT3 mutants were cultured in the presence of LIF. The mRNA and protein levels of Nanog and USP21 were analysed with PCR with reverse transcription (top) and western blotting (bottom), respectively. USP21 was detected with an anti-USP21 antibody from Abgent. Data are means±s.d. (*n*=3). **P<0.01,***P<0.001 versus vector (Student's *t*-test).

Because USP21 is required for the self-renewal of ESCs, we investigated whether its expression was regulated by signals involved in ESC maintenance. Interestingly, we found that withdrawal of LIF induced spontaneous differentiation of mESCs that was accompanied by the marked decrease of both the messenger RNA and protein levels of USP21 and the pluripotency markers Nanog, Oct4 and Sox2 (Fig. 4a,b). Similar results were obtained for the retinoic acid (RA)-induced differentiation of mESCs (Supplementary Fig. 4a,b).

To examine the mechanism by which USP21 expression was downregulated during mESC differentiation, we analysed the potential binding sites of transcriptional factors on the USP21 promoter using the Vista 2.0 software. We did not find potential binding sites of pluripotency factors, including Oct4, Nanog and Sox2. However, two potential STAT3-binding sites were identified within the −836 to −828 and −3,054 to −3,033 regions (Fig. 4c). STAT3 is a TF that is required to maintain mESCs in their undifferentiated state and is activated by LIF/JAK signalling^22^. Therefore, we examined whether the expression of USP21 was regulated by STAT3. Chromatin immunoprecipitation (ChIP) analysis revealed that STAT3 was preferentially enriched at the USP21 promoter in mESCs cultured in LIF-containing medium, and this enrichment was significantly reduced when the mESCs were cultured in medium without LIF for three days (Fig. 4c). Moreover, the expression of exogenous STAT3 effectively activated USP21 promoter-driven luciferase expression in a dose-dependent manner (Fig. 4d), whereas mutating the core STAT3-binding sequence (TGCTTCCCC to TGCCCACCC) within the USP21 promoter abolished the effect of STAT3 (Fig. 4e). Knockdown of STAT3 in mESCs also significantly reduced the messenger RNA and protein levels of USP21 (Fig. 4f). Phosphorylation at Tyr 705 (pY705) has been reported to be essential for STAT3 activation^22^. In accordance with these results, treatment with Cryptotanshinone, a specific inhibitor of STAT3 by inhibiting STAT3 Y705 phosphorylation, reduced the expression of USP21 (Supplementary Fig. 4c). Our data indicated that STAT3 (Y705D, which mimics pY705), but not STAT3 (Y705F, which cannot be phosphorylated), stimulated USP21 expression in E14 cells (Fig. 4g). The level of STAT3 pY705 was reduced upon ESCs differentiation induced by LIF withdrawal or RA treatment (Supplementary Fig. 4d,e). Together, these data indicate that USP21 is a direct transcriptional target of the LIF/STAT3 pathway in mESCs.

### USP21 phosphorylation blocks its effect on Nanog

**Figure 5:**
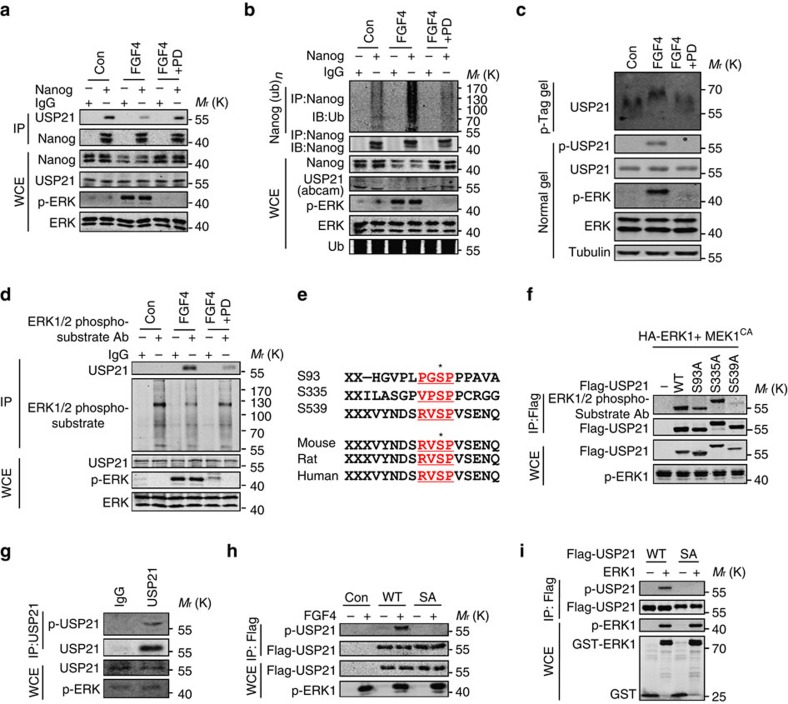
USP21 is phosphorylated by ERK1. (**a**) E14 cells were treated with mFGF4 (25 ng ml^−1^) in the presence of PD0325901 (1 μM) 12 h. Endogenous Nanog was immunoprecipitated with Nanog specific antibody, and the associated USP21 was examined by an anti-USP21 antibody from Abgent. (**b**) E14 cells were treated with mFGF4 (25 ng ml^−1^) in the presence of PD0325901 (1 μM) or not for 12 h. Endogenous Nanog was immunoprecipitated with an anti-Nanog-specific antibody, and polyubiquitination of Nanog was examined by western blotting with an anti-Ubiquitin antibody. USP21 was examined by an anti-USP21 antibody from Abgent. (**c**) E14 cells were treated with mFGF4 (25 ng ml^−1^) in the presence of PD0325901 (1 μM) or not for 12 h. Phos-Tag SDS–PAGE was applied to detect the band shift of USP21 caused by phosphorylation. USP21 was examined by an anti-USP21 antibody from Abgent. (**d**) E14 cells were treated with mFGF4 (25 ng ml^−1^) in the presence of PD0325901 (1 μM) or not for 12 h. Cell lysates were immunoprecipitated with antibody against ERK1/2 substrates and USP21 was detected with an anti-USP21 antibody. The anti-USP21 antibody is from Abgent. (**e**) Three conserved ERK1/2 phosphorylation motif ‘XXS/TP' (underlined) in USP21. The asterisk indicates the potential phosphorylation site of mouse USP21 at S93, 335 and 539. (**f**) HEK293T cells were transfected with ERK1, MEK1^CA^ and various mutants of Flag-USP21 (WT, S93A, S335A and S539A) mutants, and the Flag vector was used as control. Flag-USP21 was immunoprecipitated using the anti-Flag antibody and the phosphorylated USP21 was analysed with the anti-ERK1/2 phospho-substrate antibody. (**g**) E14 cells were treated with mFGF4 (25 ng ml^−1^) in the presence of PD0325901 (1 μM) or not for 12 h. USP21 were immunoprecipitated with an anti-USP21 antibody and then immunoblotted with an anti-phospho-USP21 antibody. The anti-USP21 antibody is from Abgent. (**h**) E14 cells stably expressing Flag-tagged WT or S539A (SA) were treated with mFGF4 (25 ng ml^−1^) in the presence of PD0325901 (1 μM) or not for 12 h. USP21 was immunoprecipitated with an anti-Flag antibody and then immunoblotted with an anti-phospho-USP21 antibody. (**i**) Phosphorylation of USP21 by ERK1 at S539 using *in vitro* kinase assay.

Extracellular-signal regulated kinase (ERK) activation leads to mESC differentiation^23^. Thus, we were interested in whether the regulation of Nanog by USP21 was influenced by ERK signalling. Our data revealed that the interaction between USP21 and Nanog was decreased upon epidermal growth factor stimulation in HEK293T cells, and that this effect was partially blocked by ERK1/2 inhibitor (SCH772984) (Supplementary Fig. 5a), suggesting that the interaction between USP21 and Nanog is regulated by the ERK pathway. Furthermore, treatment of mESCs with FGF4 (a growth factor that induces ERK activation and mESC differentiation^15^) reduced the interaction between Nanog and USP21 in mESCs in a manner that could be reversed by the MEK inhibitor PD0325901 (Fig. 5a). Consistently, Nanog ubiquitination was increased in FGF4-stimulated mESCs, which was blocked by PD0325901 (Fig. 5b). Moreover, activation of ERK1 by co-expression of a constitutively active MEK1 (MEK1^CA^) in HEK293T cells significantly impaired the interaction between USP21 and Nanog (Supplementary Fig. 5b). Interestingly, we also noticed that co-expression of MEK1^CA^ and ERK resulted in the band shift of both Nanog and USP21 (Supplementary Fig. 5b), which is possibly caused by the phosphorylation of Nanog and USP21 by the activated ERK. Furthermore, co-expression of MEK1^CA^ and ERK1 inhibited the USP21-mediated deubiquitination of Nanog (Supplementary Fig. 5c). These data indicate that the USP21-mediated deubiquitination of Nanog is regulated by ERK activation.

Next, we investigated the mechanism by which ERK1 regulates USP21-mediated Nanog deubiquitination. We first examined whether ERK1 was a binding partner of USP21 and found that exogenously expressed USP21 interacted with ERK1 in HEK293T cells, as analysed by a co-IP assay (Supplementary Fig. 5d). Next, we evaluated whether USP21 was a phospho-substrate of ERK1. We analysed USP21 phosphorylation by using a phospho-Tag gel and found that both endogenous and exogenous USP21 were significantly phosphorylated upon the activation of ERK (Fig. 5c; Supplementary Fig. 5e). By using an antibody that specifically recognizes the ERK1/2 phosphorylation motif, USP21 could be found in the immunoprecipitation (IP) products from E14 cell stimulated with FGF4. In PD0325901-treated cells, however, much less USP21 was IPed by the ERK1/2 phosphorylation motif antibody (Fig. 5d). These results confirmed that USP21 is a phospho-substrate of ERKs.

Then, we analysed potential ERK phosphorylation motifs in USP21 by using Scansite (www.scansite.mit.edu) and found that USP21 contains three putative sites with the consensus ERK phosphorylation motifs XXpS/pTP (S93, S335 and S539), with S539 conserved in mice, rats and humans (Fig. 5e). Interestingly, mutation of S539, but not the other two sites, to Ala, markedly reduced the ERK1-mediated phosphorylation of USP21 (Fig. 5f), suggesting that USP21 is phosphorylated by ERK1 at S539. To further confirm this finding, we generated an antibody that specifically recognized phospho-S539 (p-S539) in USP21 (Supplementary Fig. 5f). Treating mESCs with FGF4 significantly induced S539 phosphorylation of the endogenous USP21 or exogenously expressed WT-USP21, but not the S539A mutant (Fig. 5g,h). In an *in vitro* phosphorylation assay, WT-USP21, but not the S539A mutant, was found to be phosphorylated by ERK1 at S539, confirming that USP21 is a direct substrate of ERK1 (Fig. 5i). Collectively, these data clearly demonstrate that USP21 is directly phosphorylated by ERK1 at Ser539.

**Figure 6:**
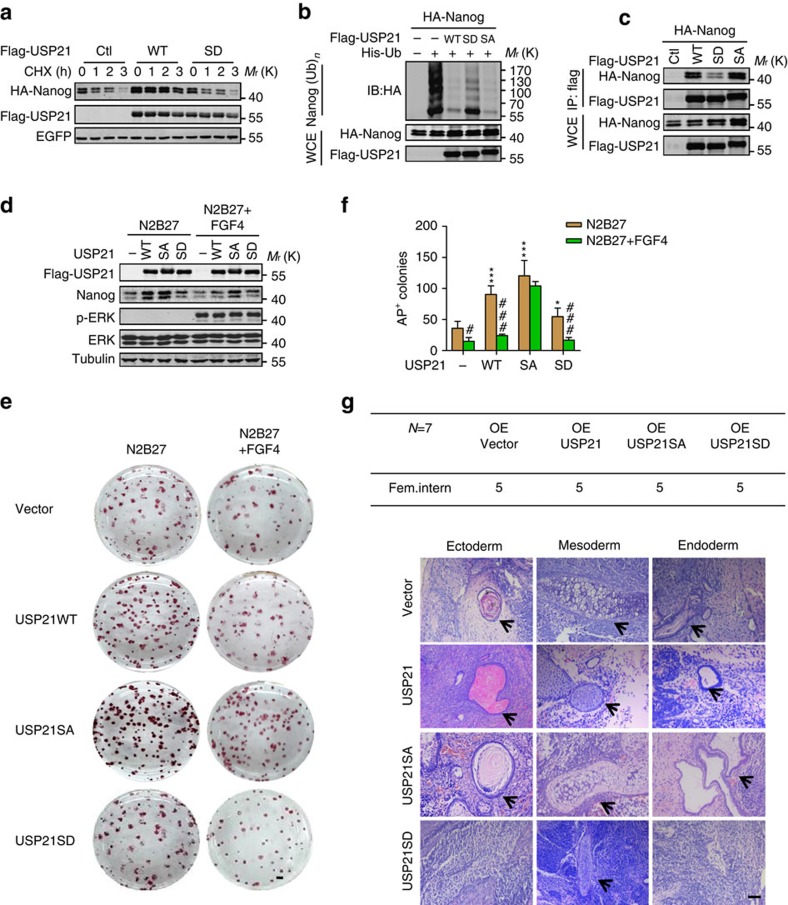
Phosphorylation of USP21 at S539 blocks its effect on Nanog function. (**a**) HA-Nanog was co-expressed with WT or S539D Flag-USP21 in HEK293T cells. The cells were treated with CHX (10 μg ml^−1^) and the protein levels of Nanog were analysed by western blotting. (**b**) HA-Nanog and His-ubiquitin were co-expressed with WT, S539D or S539A USP21 in HEK293T cells. After treatment with MG132 (10 μM) for 6 h, the ubiquitinated proteins were pulled down under denaturing conditions using Ni-NTA agarose beads and the polyubiquitination of Nanog was detected by western blotting using an anti-HA antibody. (**c**) HA-Nanog was co-expressed with WT, S539D or S539A Flag-USP21 in HEK293T cells. USP21 was immunoprecipitated with an anti-Flag antibody, and the associated Nanog was analysed by western blotting with an anti-HA antibody. (**d**) E14 cells were infected with virus containing WT, S539D or S539A Flag-USP21 for 36 h, and then treated with mFGF4 (25 ng ml^−1^) for 12 h. The protein level of Nanog was analysed by western blotting. (**e**,**f**) E14 cells were transfected Flag-USP21 WT/S539A/S539D. Thirty-six hours later, the transfected cells were cultured in N2B27 medium overnight and treated with mFGF4 (25 ng ml^−1^) for 12 h before alkaline phosphatase staining. Scale bar, 1 mm. AP-positive colonies were analysed. Data are means±s.d. (*n*=3). *P<0.05, ***P<0.001 versus USP21 (−) (two-way analysis of variance (ANOVA) test); ^#^*P*<0.05, ^###^*P*<0.001 versus N2B27 condition (two-way ANOVA test). (**g**) Haematoxylin and eosin staining of teratomas formed by E14 cells expressing WT, S539D or S539A USP21. Scale bar, 100 μm.

Next, we asked whether the phosphorylation of USP21 at S539 affected its ability to stabilize Nanog. Interestingly, we found that the WT and dephosphomimetic mutant S539A, but not the phosphomimetic mutant S539D, significantly stabilized Nanog (Fig. 6a; Supplementary Fig. 6a). Concordantly, the USP21 S539D mutant also partially lost the ability to deubiquitinate Nanog (Fig. 6b). We found that S539D, but not S539A, S93D or S335D, showed a reduced ability to interact with Nanog (Fig. 6c; Supplementary Fig. 6b). In mESCs, both the MEK inhibitor PD0325901 and ERK1/2 inhibitor SCH772984 significantly stabilized Nanog (Supplementary Fig. 6c). Overexpression of both the WT-USP21 and S539A mutant, but not the S539D mutant, stabilized Nanog (Fig. 6d) and promoted self-renewal of mESCs in normal culture condition (Supplementary Fig. 6d). More significantly, S539A mutant almost completely prevented mESC differentiation induced by FGF4 (Fig. 6e,f). Furthermore, compared with the WT and S539A mutant USP21, mESCs with overexpression of S539D displayed a reduced ability to form teratomas *in vivo* (Fig. 6g; Supplementary Fig. 6e). Collectively, these data demonstrate that ERK1-induced phosphorylation of USP21 at S539 prevents its interaction with Nanog and thus negatively regulates USP21-mediated Nanog deubiquitination and stabilization.

### USP21 regulates H2A ubiquitination via Nanog

USP21-LV is a shuttling protein that is primarily localized in the cytosol because it contains a nuclear export signal at its N terminus^24^. Interestingly, we found that both isoforms of USP21 were re-localized into the nucleus when co-expressed with Nanog in HeLa cells (Supplementary Fig. 7a). These results intrigued us to examine whether USP21 has other functions in the nucleus by interacting with Nanog. USP21 has been reported to deubiquitinate the monoubiquitination of H2A at K119 (H2AK119Ub1)^25^, this deubiquitination is required for polycomb repressive complex 1-mediated gene repression and plays critical roles in maintaining the stemness of ESCs^26^,^27^,^28^. Thus, we hypothesized that Nanog might recruit USP21 to gene promoters where it deubiquitinates H2A and regulates gene expression.

**Figure 7:**
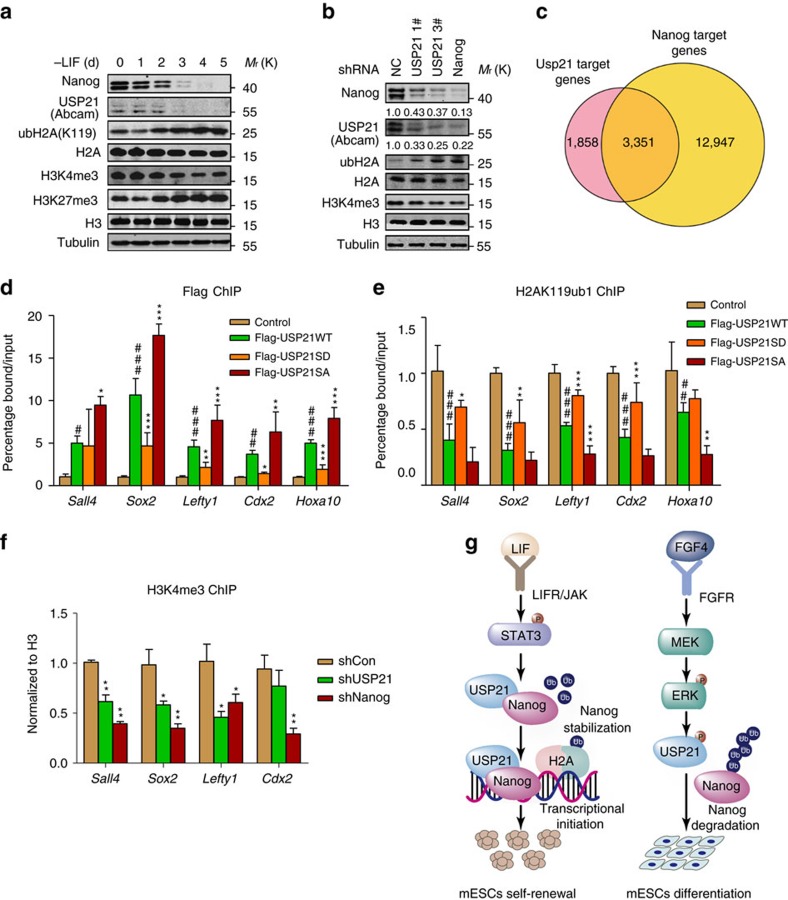
USP21 is recruited to gene promoters and modulates H2A ubiquitination by interaction with Nanog. (**a**,**b**) Histone ubiquitination and methylation were analysed in E14 cells cultured in LIF-free condition (**a**) or in E14 cells stably expressing the control or shRNA targeting USP21 or Nanog (**b**). The anti-USP21 antibody is from Abcam. (**c**) Venn diagram depicting overlap between target genes of Usp21 and Nanog defined by their binding peaks (see the ‘ChIP-Seq data analysis' in the Methods section). Nanog ChIP-Seq data are referred to GEO Data Sets with project number GSE44764. (**d**) Binding of USP21 and its mutants to the Nanog-regulated genes. Data are means±s.d. (*n*=3). ^#^*P*<0.05, ^##^*P*<0.01, ^###^*P*<0.001 versus control (Student's *t*-test); *P<0.05, **P<0.01, ***P<0.001 versus USP21 WT (Student's *t*-test). (**e**) The effect of USP21 and its mutants on the H2A ubiquitination at K119 at Nanog-regulated genes. Data are means±s.d. (*n*=3). ^##^*P*<0.01, ^###^*P*<0.001 versus control (two-way analysis of variance (ANOVA) test); *P<0.05, **P<0.01, ***P<0.001 versus USP21 WT (two-way ANOVA test). (**f**) USP21 affects the H3K4me3 level at Nanog-regulated genes. Data are means±s.d. (*n*=3). *P<0.05, **P<0.01 versus shCon (two-way ANOVA test). (**g**) Model showing that USP21 plays an important role in the maintenance of mESC self-renewal through regulating ubiquitination of Nanog and ubH2A.

To test this hypothesis, we examined whether the level of H2AK119Ub1 was affected during mESC differentiation. H2AK119Ub1 was significantly increased in mESCs cultured under LIF-free conditions and was accompanied by the downregulation of USP21 and Nanog. The monoubiquitination of H2A at K119 has been reported to reduce the level of H3K4me3 (ref. 25). In agreement with this observation, we found that the level of H3K4me3 was decreased, and that of H3K27me3 increased during in mESCs cultured in LIF-free medium (Fig. 7a). Similar results were obtained in RA-induced differentiation of mESCs (Supplementary Fig. 7b). We also found that H2AK119ub1 was increased with the reduction of H3K4me3 upon USP21 or Nanog knockdown in mESCs (Fig. 7b).

Next, we tested whether the monoubiquitination of H2A at K119 was regulated by USP21 in mESCs. E14 cell lines expressing Flag-USP21 (WT, S539A or S539D) were generated by insertion of USP21 WT or mutants at the Rosa26 locus with CRISPR/Cas9 and homologous recombination (Supplementary Fig. 7c). Cells expressing WT or S539A USP21, but not S539D USP21, showed significantly reduced levels of H2AK119ub1 (Supplementary Fig. 7d). Genome-wide USP21 and Nanog ChIP sequencing (ChIP-Seq) revealed that ∼60% of USP21 target genes were co-bound by Nanog (Fig. 7c). These data suggest that Nanog is an important and major cofactor of USP21 in mESCs. These data also suggested that USP21 and Nanog might independently regulate a number of other genes. ChIP–quantitative PCR (qPCR) analysis using an anti-Flag antibody indicated that Flag-USP21 was significantly enriched at Nanog-regulated genes, such as *Sall4*, *Sox2*, *Lefty1*, *Cdx2* and *Hoxa10* (refs 29, 30) (Fig. 7d). Furthermore, we observed decreased enrichment of ubH2A and increased enrichment of H3K4me3 in the Flag-USP21 expressing mESCs compared with the parental control cells (Fig. 7e; Supplementary Fig. 7e). Conversely, depletion of USP21 or Nanog decreased the enrichment of H3K4me3 to gene promoters (Fig. 7f). Together, these results indicate that in addition to stabilizing Nanog, USP21 is recruited to chromatin by Nanog to deubiquitinate H2A and facilitate Nanog-mediated gene expression in mESCs.

## Discussion

Post-translational protein modifications (PTMs) play important roles in controlling the activity, interactions, subcellular localization and stability of their target proteins. Nanog is a critical TF that controls the pluripotency of ESCs and early embryonic development. However, its regulations by PTMs are not well understood. The most well-studied Nanog PTM is phosphorylation^31^. Several Nanog phosphorylation sites have been revealed, and several kinases, including focal adhesion kinase, protein kinase C, ERKs and CDK1, have been reported to phosphorylate Nanog. The phosphorylation of Nanog has been reported to be involved in ESC self-renewal and carcinogenesis^17^,^32^,^33^,^34^,^35^.

Ubiquitination is another important form of PTM mediated by ubiquitin ligases and DUBs that regulate protein functions and stability. The protein levels of key pluripotency factors are controlled by the UPS. For example, Oct4 is regulated by Itch, which is a C2-WW-HECT domain E3 ligase, in undifferentiated mESCs^36^. Oct4 and Sox2 are regulated by the E3 ligase WWP2 during differentiation^37^,^38^. Klf4 is degraded by E3 ubiquitin ligase β-TrCP1 and β-TrCP2 (refs 28, 39). c-Myc is degraded by E3 ligase Fbxw7 during ESC differentiation^40^ and Rex1 is degraded by E3 ligase RNF12 (ref. 41). Although several ubiquitin E3 ligases of stem cell transcription factors (SCTFs) have been identified, little is known about the mechanism and function of the deubiquitinating enzymes that controls the protein levels of these key pluripotency factors in ESC differentiation and function. In this study, we identified USP21 as a DUB that specifically regulates Nanog stability and function in mESCs.

On the basis of our studies and previous studies by other groups, we propose that each SCTF is regulated by both ubiquitination and deubiquitination at the post-translational level. The net balance of the ubiquitination and deubiquitination of SCTFs could have a significant impact on the cell fate determination of stem cells. Increased ubiquitination of SCTFs leads to degradation and induces cell differentiation, whereas dominant deubiquitination of SCTFs stabilizes these TFs, thus promoting the maintenance of stem cells. Our studies also suggest that the protein stability of each SCTF might be controlled by at least a pair of specific DUB and ubiquitin E3 ligase. Therefore, dissecting this paradigm of reciprocal post-translational control, especially ubiquitination and deubiquitination, in stem cell regulatory networks not only advances stem cell biology but also promotes understanding of cancer stem cells.

USP21 can deubiquitinate both the nuclear and cytoplasmic proteins, such as GATA3, RIPK1 and RIG-1 (refs 42, 43, 44). USP21 functions as a negative regulator in antiviral responses through binding and deubiquitinating RIG-1 in the cytosol^44^. USP21 regulates centrosome- and microtubule-associated functions^18^,^45^. Moreover, USP21 affects the transcription of NF-κB p65 through deubiquitinating and stabilizing interleukin-33 in the nucleus^46^. In this study, we provided evidence showing that USP21 specifically deubiquitinated and stabilized Nanog, but not other pluripotency factors (for example, including Sox2 and Oct4) in mESCs. Consequently, USP21 was required for mESC maintenance. Depletion of USP21 promoted the differentiation of mESCs and reduced the efficiency of somatic cell reprogramming. Thus, our study uncovers an essential function of USP21 in mESC stemness maintenance.

We found that USP21 is regulated at both transcriptional and post-translational levels in mESC to regulate Nanog function. At the transcriptional level, the expression of USP21 in mESCs was activated by the LIF/STAT3 pathway, which was critical for the maintenance of mESC and the self-renewal of mESCs. Upon mESC differentiation, the expression of USP21 was significantly downregulated. At the post-translational level, USP21 was phosphorylated by ERKs induced by differentiation cues. This phosphorylation reduced the binding of USP21 to Nanog and led to Nanog degradation. These data suggest that regulation of Nanog by USP21 is a precise and important event to determine the mESC fate.

The ERK signalling pathway has been shown to negatively regulate the stability of several pluripotent factors. For example, ERKs phosphorylate Klf4 and induce Klf4 degradation by promoting its binding to β-TrCP1 or β-TrCP2 (ref. 47), ERK2 also negatively regulates the stability of Klf2 (ref. 48). In addition, Nanog is a target of ERK1 and phosphorylation by ERK1 induces Nanog degradation via the E3 ligase FBXW8 (ref. 17). Although it is well established that the LIF–STAT3 pathway is essential for the maintenance of mESC, it is also reported that LIF can stimulate the ERK pathway and promote mESCs differentiation. Inhibition of ERK activation enhances mESCs response to LIF and maintains mESCs self-renewal^49^. Here we demonstrated that ERK1 regulated Nanog stability by phosphorylating its DUB USP21 at S539, which impaired the interaction between USP21 and Nanog, and led to increased ubiquitination and degradation of Nanog. All these studies strongly support that ERK signalling is an essential negative regulatory mechanism that controls mESC stemness at multiple levels.

USP21 has been reported to deubiquitinate histone H2A and activate transcriptional initiation via trans-histone cross-talk with H3K4 di- and trimethylation during hepatocyte regeneration^25^. H2A ubiquitination and chromatin compaction combine to mediate the polycomb repressive complex 1-dependent repression of genes that are crucial for the maintenance of ESC identity^50^. However, limited information is available concerning the role of USP21 in the regulation of histone function and gene expression. We demonstrated that USP21 not only deubiquitinated Nanog by direct interaction but also regulated H2A ubiquitination through being recruited to histones by Nanog.

Above all, our data demonstrate that USP21 is necessary to maintain the self-renewal of mESCs, and that depletion of *Usp21* leads to enhanced differentiation. These results indicate that *Usp21* depletion may result in abnormality in development. It is most surprising to note that a recent report showed that *Usp21*-KO mice developed normally and did do not show obvious phenotypes^51^. The discrepancy is probably caused by the compensation of other DUBs for Nanog during early development in the *Usp21*-KO mice. A recent study has shown that *Egf17* knockdown leads to the abnormal development of blood vessels, whereas *Egf17*-KO does not affects blood vessel growth; these results indicate knockdown or KO of the same gene might lead to different consequences^52^. It has been reported that transient downregulation of Nanog in mESCs appears to direct cells towards differentiation, but mESCs with genetic deletion of *Nanog* can self-renew indefinitely, although they are more prone to differentiation^53^. This is consistent with the report showing that *Stat3*-null mESCs can also self-renewal under 2i culture conditions^15^. However, these *Nanog*-KO mESCs failed to form mature germ cells^53^. Another report has found that, although contributing greatly to reprogramming efficiency, *Nanog* is not absolutely required for the generation of iPSCs, and the *Nanog-*null iPSCs can contribute to mature germ cells^54^. Another possibility is that Nanog is only transiently required and is quickly degraded around the time of implantation^2^. These somewhat conflicting results indicating more studies are necessary to fully elucidate the functions of Nanog in pluripotency control and development. Thus, although we have shown that the stabilization of Nanog by USP21 is required for the long-term maintenance of mESCs *in vitro*, their *in vivo* regulations remain to be discovered.

In summary, we identified USP21 as a specific deubiquitinating enzyme for the pluripotency factor Nanog. USP21 plays a critical role in maintaining the self-renewal of mESCs by stabilizing Nanog and facilitate the chromatin remodelling that favors Nanog-mediated gene transcription. Depletion of USP21 leads to Nanog degradation and mESC differentiation. The regulation of Nanog by USP21 is controlled by both LIF/STAT3 and FGF/ERK signalling pathway: the expression of USP21 is regulated by LIF/STAT3 signalling and the function of USP21 is regulated by ERKs. Our study suggests a regulatory mechanism by which extrinsic signals regulates the fate of mESCs via the deubiquitination of pluripotency factor (Fig. 7g). The dynamic reversibility of the ubiquitin modification and the recent development of general proteasome inhibitors^55^ and specific blockers and activators of ligase functions^56^,^57^ pave the way for the future manipulation of the UPS in ESCs. In particular, this approach may lead to more efficient reprogramming of somatic cells or provide new therapies targeting cancer stem cells.

## Methods

### Antibodies and reagent

Antibodies were obtained from commercial sources: the anti-HA, anti-Flag and secondary antibodies were obtained from Sigma. The anti-ubiquitin (P4D1 sc-8017, 1:1,000), anti-H2A (L2313, 1:1,000) and anti-H2B (C2312, 1:1,000) were obtained from Santa Cruz Biotechnology (Santa Cruz, CA, USA). The antibodies against STAT3 (9139S, 1:1,000), P44/42 MAPK (9194S, 1:2,000), p-P44/42 MAPK (4370P, 1:500), p-MAPK/CDK substrates (2325S, 1:500), H3 (4620S, 1:5,000), H3K4me3 (9751S. 1:1,000) and H3K27me3 (9733S, 1:1,000) were obtained from CST. The anti-Oct4 (ab19857, 1:5,000) and anti-USP21 (ab171028, 1:500) antibodies were from Abcam. The antibody against Nanog was from Bethyl (A300-397A, 1:2,000) and CST (8822, 1:1,000). The anti-tubulin (1:3,000) antibody was kindly provided by Dr Dianqing Wu. The antibody against GAPDH (2118s, 1:10,000) was from CST. The anti-p-STAT3 (Y705; 2236-1, 1:1,000) and anti-Sox2 (2683-1, 1:3,000) were from Epitomics. The antibodies against ubH2A (K119; 05-678, 1:500) and ubH2B (K120; 05-1312, 1:500) were purchased from Millipore. Glutathione SepharoseTM 4B (17-0756-01) was obtained from GE Healthcare. The NI-NTA Agarose (30210) was purchased from Qiagen. The rabbit polyclonal antibody against phospho-USP21 was generated using a CNDSRVPp[Ser]PVSEN peptides (Abmart, 1:500). The rabbit polyclonal antibody against USP21 was generated using a CNDSRVSPVSEN peptides by Abgent (1:500). DMEM (amino acid free) was purchased from Genetimes Technology, and fetal bovine serum (FBS; 10099), GlutaMAX (100 × ; 1293468), non-essential amino acids (NEAA; 100 × ; 11140), β-mercaptoethanol (741953), penicillin and streptomycin (15140) were purchased from Gibco. The LIF (ESG1107) and RA (554723) were from Millipore. MG132 (10 μΜ) and cycloheximide (10 μΜ) were from Sigma. MEK inhibitor PD0325901 (1 μΜ) was from Sigma, ERK1/2 inhibitor SCH772984 (10 μΜ) and STAT3 inhibitor xryptotanshinone (10 μΜ) were from Selleck. Recombinant mouse fibroblast growth factor-4 (25 ng ml^−1^) was from R&D and epidermal growth factor (100 ng ml^−1^) was from Sigma.

### Cell culture and derivation of MEFs

Human embryonic kidney 293T cells (American Type Culture Collection), Henrietta Lacks strain of cancer cells (HeLa cells, American Type Culture Collection) and MEF cells were cultured in DMEM supplemented with 10% heat-inactivated fetal bovine serum at 37 °C in 5% CO_2_. OG2 mice, which carry the Rosa26-lacZ allele and a transgenic Oct4 promoter-driven GFP expression, were mated with C57 mice. MEFs were isolated from E12.5 embryos. Gonads and internal organs were removed before MEF isolation. MEFs were grown in DMEM supplemented with 10% (vol/vol) FBS, 2 mM L-glutamax, 0.1 mM NEAAs, 100 U ml^−1^ penicillin and 100 μg ml^−1^ streptomycin. Isolated MEFs in passage 1 were used for further experiments. E14 cells were maintained feeder free on gelatin-coated plastics in mES media (DMEM with 15% FBS, 2 mM GlutaMAX, 0.1 mM NEAAs, 0.1 mM β-mercaptoethanol, 100 U ml^−1^ penicillin and 100 μg ml^−1^ streptomycin supplemented with 1,000 U ml^−1^ LIF and passaged every 3 days.

### Cell transfection

Transfections were carried out by SunbioTrans-EZ for HeLa cells, using calcium phosphate–DNA coprecipitation for HEK293T cells, using X-tremeGENE HP and Lipofectamine 2000 for E14 cells according to the manufacturer's instructions.

### Animal procedures

Male 6-week-old BALB/cA nude mice used in the present study were purchased from National Rodent Laboratory Animal Resources (Shanghai, China). *Usp21*^Floxp/Floxp^ mice in a C57BL/6N background were purchased from the Jackson Laboratory (MECN EPD0716_10_H09; Taconic, USA). Animals were caged in the groups of five in a laminar airflow cabinet under specific pathogen-free conditions, fed with sterilized food and water, and kept on a 12-h light/dark cycle. All treatments were administered according to the guidelines of Institution Animal Care and Use Committee and all the protocols were approved by East China Normal University.

### Teratoma formation

Approximately 1 × 10^6^ E14 cells were suspended in 200 μl of mES medium and injected into the thigh muscle of male 6-week-old BALB/cA nude mice. The animals were checked two to three times per week. Five to six weeks after injection, the teratomas were permitted to grow to 15 mm in any direction by the Institutional Animal Care and Use Committee at Massachusetts General Hospital and collected, fixed overnight with 4% paraformaldehyde, embedded in paraffin and sectioned. Sections of the teratomas were stained with haematoxylin and eosin, and analysed histologically.

### Derivation and characterization of ESCs

Blastocysts were flushed out from the uterus of *Usp21*^Floxp/Floxp^ and WT mice E3.5 female mice with a 27.5-G needle connected to 1-ml syringe filled with 1 ml M2 medium. Blastocysts were then transferred to matrigel-coated plates with 200-ml sterile tip and cultured with M2 medium or regular ESC medium for 3–5 days. ESC-like colonies formed from inner cell mass (ICM) outgrowths were handpicked using glass needle and transferred to plates coated with a feeder layer. Several days later, ESC-like colonies were dissociated by 0.25% trypsin and expanded in ESC medium. Genotyping to detect the presence of the *USP21*^Loxp^ used the following primer sequences: *USP21*_55571_F: 5′-GGTGAGGACTTGACAGCACA-3′; *USP21*-AS1: 5′-CCTAAGGACCCAAGGAAGAA-3′; and *USP21* Mutant-AS1: 5′-AACTACAAGGGGATCCCAAG-3′.

### Plasmids and virus

DUBs plasmids were obtained from Addgene. Other constructs were generated by standard molecular cloning method. The MEK1^CA^ was kindly provided by Dr MienChie Hung. The NANOG reporter plasmids were kindly provided by Dr Duanqing Pei^58^. For the NANOG reporter plasmids, oligonucleotides containing the NANOG-binding site (5′-cccttcgccgattaagtacttaag-3′) and a SalI restriction site were chemically synthesized (sense:5′-tcgacacccttcgccgattaagtacttaag-3′; antisense: 5′-tcgacttaagtacttaatcggcgaagggtg-3′). After being 5′-end phosphorylated by T4 kinase and ATP, these oligonucleotides were annealed and ligated to the SalI site of p37tk-luciferase. Positive clones were randomly picked, and the copy numbers of the inserted NANOG-binding site were determined by sequencing. Nanog, OCT4, Sox2, Klf4, ERK1, USP21 and its mutants, USP2, STAT3 and its mutants were cloned into the pCDNA3.1 vector that a Flag or HA tag was fused to the N terminus using standard cloning methods. USP21 basic promoter luciferase reporter and its mutants were cloned into the PGL3 basic vector. GST-tagged ERK1, GST-tagged USP21 and its mutants were cloned into pGEX-4T-1. His-tagged Nanog was cloned into pET28a. Nanog-luciferase-IRES-Renilla was cloned into pCDNA3.1 vector. To generate lentivirus, USP21 and its mutants, and Cre were cloned into the pWPI lentiviral vector. shRNAs were cloned into the FG12 vector. All the vectors were confirmed using DNA sequencing.

### Short hairpin RNA design

Target sequences: *USP21* shRNA1: 5′-CCCACTTTGAGACGTAGTA-3′; *USP21* shRNA2: 5′-CTCCTGTGAAGCTGTGAAT-3′; *USP21* shRNA3: 5′-GACCGAGCCAACTTAATGT-3′; *USP21* shRNA4 (target *USP21* 3′UTR): 5′-GTCCCATGTACAGAAACCA-3′; *USP2* shRNA: 5′-GCGAATGGCACACTTTCAA-3′; *Nanog* shRNA: 5′-GCCAACCTGTACTATGTTTAA-3′; Scramble shRNA: 5′-TTCTCCGAACGTGTCACGTTT-3′; *STAT3* siRNA1: 5′-GACCAGCAATATAGCCGAT-3′; *STAT3* siRNA2: 5′-GGGTCTCGGAAATTTAACA-3′; *STAT3* siRNA3 (target STAT3 3′UTR): 5′-GCACTTTCAACCTTGCTAA-3′.

### Virus preparation and viral infection

To prepare lentivirus for the knockdown experiments, HEK293T cells were transduced with the FG12 vector and the lentivirus packaging vectors PHCMVG, PMDLg/PRRE and PRSV-Rev using the calcium phosphate–DNA co-precipitation method. To prepare lentivirus for protein expression, the HEK293T cells were transduced with pWPI vectors and the packaging vectors PSPAX2 and pMD2G using the calcium phosphate–DNA co-precipitation method. Medium containing the virus was collected 48 h after transfection. The E14 cells were cultured in the collected viral supernatant in the presence of polybrene (8 μg ml^−1^).

### Luciferase reporter assay

HEK293T cells were transiently co-transfected with firefly luciferase reporter vectors, effector vectors and the renilla luciferase vector. After 36 h, cells were collected in lysis buffer (25 mM Tris-Cl (pH 7.8), 25 mM dithiothreitol (DTT), 2 mM 1,2-diaminocyclo-hoxane *N*,*N*,*N*,*N*′-tetracetic acid, 10% glycerol and 1% Triton X-100), and luciferase assays were performed using the dual-luciferase reporter assay system (Promega).

### Immunoprecipitation and western blotting

Transfected HEK293T cells were lysed in lysis buffer (50 mM Tris-Cl, pH 7.4, 0.5% NP-40, 150 mM NaCl, 1 mM EDTA, 10% glycerophosphate and a cocktail of proteinase inhibitors). After lysis for 30 min, the soluble fraction of the cell lysates was isolated via centrifugation at 12,000 r.p.m. in a microcentrifuge for 15 min at 4 °C. For IP, the cell lysates were centrifuged to remove the cell debris and were incubated in HA-conjugated beads (Abmart) or M2 beads (Sigma) for 2–3 h. Endogenous Nanog was immunoprecipitated using an anti-Nanog polyclonal antibody. The beads were boiled after extensive washing; the proteins were boiled, resolved via SDS–polyacrylamide gel electrophoresis (SDS–PAGE) gel electrophoreses and analysed via immunoblotting. The proteins were detected using the Odyssey system (LI-COR Biosciences). All uncropped western blots can be found in Supplementary Figs 8–10.

### Ubiquitination assay

For the *in vivo* ubiquitination assay using Ni-NTA beads, the cells were transfected with His-ubiquitin. Then, the transfected cells were lysed using denaturing buffer A (6 M guanidine-HCl, 0.1 M Na_2_HPO_4_/NaH_2_PO_4_, 10 mM imidazole, pH 8.0), and the ubiquitinated proteins were purified using Ni-NTA beads. The beads were then washed sequentially with buffer A, buffer B (8 M urea, 0.1 M Na_2_HPO_4_/NaH_2_PO_4_, pH 8.0, 0.01 M Tris-HCl, pH 8.0, 10 mM β-mercaptoethanol) and buffer C (8 M urea, 0.1 M Na_2_HPO_4_/NaH_2_PO_4_, pH 6.3, 0.01 M Tris-HCl, pH 6.3, 10 mM β-mercaptoethanol) containing 0.2% Triton X-100, and buffer C. The washed beads were incubated in 40 μl of elution buffer (200 mM imidazole, 0.15 M Tris-HCl, pH 6.7, 30% glycerol, 5% SDS, 0.72 M β-mercaptoethanol) at room temperature for 30 min. The input fractions and eluates were analysed via western blotting. For endogenous ubiquitinated Nanog, E14 cells were lysed using RIPA lysis buffer (50 mM Tris-HCl, PH 7.4, 150 mM NaCl, 1% NP-40, 0.5% sodium deocycholate, 1% SDS, 1% Triton X-100). After lysis for 30 min, the soluble fraction of the cell lysates was isolated via centrifugation at 12,000 r.p.m. in a microcentrifuge for 15 min at 4 °C. For IP, the cell lysates were centrifuged to remove the cell debris and were incubated with anti-Nanog polyclonal antibody or 2–3 h. The beads were boiled after extensive washing; the proteins were boiled, resolved via SDS–PAGE gel electrophoreses, and analysed via immunoblotting.

To detect the *in vitro* deubiquitination of Nanog, His-USP21 was purified from *Escherichia coli*. The *in vitro* deubiquitination reaction was performed using Flag-Nanog, His-USP21, 50 mM Tris-Cl (PH 8.0), 50 mM NaCl, 1 mM EDTA, 10 mM DTT and 5% glycerol in a final volume of 25 μl at 37 °C for 4 h. The ubiquitinated Nanog proteins were detected via western blotting using an anti-Flag antibody.

### GST pull-down assay

The His-Nanog protein was purified from *E. coli* and incubated in 10 μg of purified GST or GST-USP21 protein. The GST proteins were purified using glutathione sepharose 4B, and the bound Nanog was detected via western blotting.

### *In vitro* phosphorylation assay

Flag-USP21 and Flag-USP21 S539A were expressed in HEK293T cells and purified using M2 beads for immunoprecipitation. After washing twice with kinase buffer (25 mM Tris-HCl (pH 7.5), 5 mM glycerophosphate, 10 mM MgCl_2_, 2 mM DTT and 0.1 mM Na_3_VO_4_), purified Flag-USP21 or Flag-USP21SA were incubated with 2 μg GST-ERK1 in 25 μl of kinase buffer containing 200 mM ATP at 37 °C for 1 h. The reaction was terminated by the addition of 8 μl of 4XLaemmli SDS sample buffer. The reaction mixtures were boiled and separated by SDS–PAGE and phosphorylation USP21 was immunobloted by anti-phosphor-USP21 antibody.

### Immunofluorescence

Cells were seeded on fibronectin-coated glass coverslips in 24-well tissue culture plates. After transfection or infection, the cells were rinsed once with PBS and fixed in 4% paraformaldehyde for 15 min at room temperature. The fixed cells were permeabilized using 0.1% Triton X-100 and rinsed twice with PBS. The coverslips were blocked with blocking buffer for 1 h (0.3% bovine serum albumin in PBS) and incubated in a primary antibody in blocking buffer overnight at 4 °C. Next, the coverslips were rinsed twice with blocking buffer and incubated in secondary antibodies for 1 h at room temperature in the dark, followed by tyramide signal amplification. The glass coverslips were mounted using Mowiol and were examined using a Zeiss LSM 510 Meta confocal system.

### Mouse iPSC generation

Retrovirus were produced by transfection of plat-E cells with pMXs retroviral vectors containing the coding sequences of mouse Oct4, Sox2, Klf4, Nanog, Nanog K138/K169R mutant, USP21-SV, USP21-LV and USP21-C221A. MEFs were seeded at a density of 150,000 cells per well in a six-well plate 18 h before infection. Virus containing supernatants, supplemented with 4–8 μg ml^−1^ polybrene, were added onto the plates of MEF cultures and spun at 2,500 r.p.m. for 90 min to ensure their infection. Medium was changed immediately after virus transduction. Two days post virus infection, MEFs were digested into single cells and reseeded at a density of 5,000 or 10,000 cells per well on 24-well plates pre-seeded with irradiated MEF feeders, supplemented with mES medium (DMEM supplemented with 15% FBS, 2 mM L-glutamax, 0.1 mM NEAA, 0.1 mM β-mercaptoethanol 1,000 U ml^−1^ LIF, 100 U ml^−1^ penicillin and 100 μg ml^−1^ streptomycin). At day 6, culture medium was replaced with KSR medium (KO DMEM supplemented with 15% KO serum replacement, 2 mM L-glutamax, 0.1 mM NEAAs, 0.1 mM β-mercaptoethanol, 1,000 U ml^−1^ LIF, 100 U ml^−1^ penicillin and 100 μg ml^−1^ streptomycin). Images of representative wells were taken, and AP-positive colonies or GFP-positive colonies were counted at day 12 or day 16 post infection.

### Real-time qPCR

Total RNA was Trizol-extracted, column-purified and reverse-transcribed using PrimeScript 1st Strand Cdna Synthesis kit (Takara). Primer sequences used to test gene expression are available in Supplementary Table 1. ChIP was performed according to the manufacturer's instructions (CST, #9004). For ChIP–qPCR analysis, 1 ng ChIP DNA was used for each PCR. All qPCR analyses were performed using Fast SYBR Green (Takara). Primer sequences are available in Supplementary Table 2.

### ChIP sequencing

*Library preparation*. An amount of 5–10 ng of ChIP DNA was used for the generation of libraries for deep-sequencing using the NEXTflex ChIP-Seq kit. In brief, the DNA was end-repaired following adding an A-base to the end-repaired DNA fragments. Illumina adaptors (regular or multiplex) were ligated to the ChIP DNA fragments and 100–300 bp of size fractions were excised from 2% agarose gel. Adaptor-modified fragments library preparation was validated in bioanalyser for quantity and size before putting into sequencing (The Beijing Genomics Institute).

### ChIP-Seq data analysis

All fastq files including previously published samples were aligned to the Mouse reference genome (UCSC, mm10 assembly) using Bowtie (settings: -p 20 -S -m 1). Only uniquely mapped reads were retained for analysis in this study. Peak detection was performed using the MACS version 2 software^59^ with default settings (except: ‘--broad --format=BAM -g mm'). The ‘—broad' parameters causes MACS to output both ‘normal' and ‘broad' peaks, where normal peaks are merged into broad peaks if they are closely located on the same chromosome. All the analyses were based on broad peaks. IgG samples were used as peak calling controls. Chromosomal positions were annotated to the RefSeq database (mm9) using the UCSC refFlat table^60^. To avoid redundancy, only the longest transcript of each gene was used. All chromosomal interval overlaps were performed using ‘intersectBed' script from UCSC. To create a bigwig (that is, wiggle format) file, each mapped read was first extended in the 3′-direction to a total length of 250 bases (our estimated fragment length). The modified alignment file was then transformed to a bedGraph file, where densities were scaled according to millions of uniquely mapped reads, before finally being converted to a bigwig file. All the target genes were analysed by the BETA-minus of cistrome. Peaks considered to contribute to the genes: 10,000 bp; the distance from gene transcription start site within which peaks will be selected: 100,000 bp. The *USP21* peaks were used as input to Cistrome BETA-minus tool with default settings to predict the target genes. The target genes of Nanog were predicted the same way. The ChIP-Seq data have been submitted to the Gene Expression Omnibus (GEO) database (GSE79890). The Nanog-binding site used to test in our study are showed in Supplementary Table 3. Nanog ChIP-Seq data refer to GEO DataSets under accession code GSE44764 (ref. 61).

### Generation of the Flag-USP21 stable cell line

Flag-USP21 WT/SD/SA mESC stable cell line was generating using CRISPR-CAS methods^62^. In brief, guide RNA (gRNA) expression vectors were constructed for pGS3-T7-gRNA. The sequence of gRNA (target to Rosa26) is 5′-TCGTGATCTGCAACTCCAG-3′. Flag-USP21 was ligated to the Sall and MluI site of Rosa26-puro-CAG donor vector. Then, we inserted the Flag-USP21 into the mouse Rosa26 safe harbour site via homologous recombination. An amount of 40 μg donor vector was electroporated into 0.3–0.4 million E14 cells along with 5 μg each of the Rosa26 gRNA and pCas9-GFP plasmid (Amaxa kit R, program A-24; Lonza). One week after electroporation, cells were replated into 150-mm dishes at clonal density. Individual colonies were subsequently selected under the fluorescence microscope and expanded in 24-well dishes. Analysis of Flag-USP21 stable cell line by western blotting.

### ESC differentiation assays

The differentiation of E14 cells was induced by the withdrawal of LIF or treatment with 10 μM RA.

### MEK–ERK pathway stimulation in mESCs

E14 cells were cultured in N2B27 medium overnight and then treated with 25 ng ml^−1^ mFGF4 for 12 h before collecting for immunoblotting analysis.

### Alkaline phosphatase

For AP staining, the cells were fixed with 4% paraformaldehyde in PBS for 30 s and rinsed once with PBS. Detection was performed using a leukocyte AP kit (Sigma, catalogue no 85L3R) according to the manufacturer's protocol.

### Statistical analysis

Statistical analyses were performed with a two-tailed unpaired Student's *t*-test and experiments that contain multiple samples were performed with analysis of variance followed by a multiple comparisons test. The data are presented as the means±s.d. The mean is calculated from truly independent experiments. The values of *P*<0.05 were considered statistically significant.

### Data availability

The authors declare that all data supporting the findings of this study are available within the article and its Supplementary Information files or from the corresponding author on reasonable request. *Usp21* ChIP-Seq data have been deposited in the NCBI GEO database under accession code GSE79890 (https://www.ncbi.nlm.nih.gov/geo/query/acc.cgi?acc=GSE79890).

## Additional information

**How to cite this article:** Jin, J. *et al*. The deubiquitinase USP21 maintains the stemness of mouse embryonic stem cells via stabilization of Nanog. *Nat. Commun.* **7,** 13594 doi: 10.1038/ncomms13594 (2016).

**Publisher's note**: Springer Nature remains neutral with regard to jurisdictional claims in published maps and institutional affiliations.

## Supplementary Material

Supplementary Information Supplementary Figures 1-10, Supplementary Table 1-3.
