# Supplementary material for: The deubiquitinase USP21 maintains the stemness of mouse embryonic stem cells via stabilization of Nanog
Source: Nat Commun. 2016 Nov 25;7:13594. doi: 10.1038/ncomms13594 (PMC5133637; doi:10.1038/ncomms13594)
Supplement: Supplementary Information — Supplementary Figures 1-10, Supplementary Table 1-3. [file ncomms13594-s1.pdf]

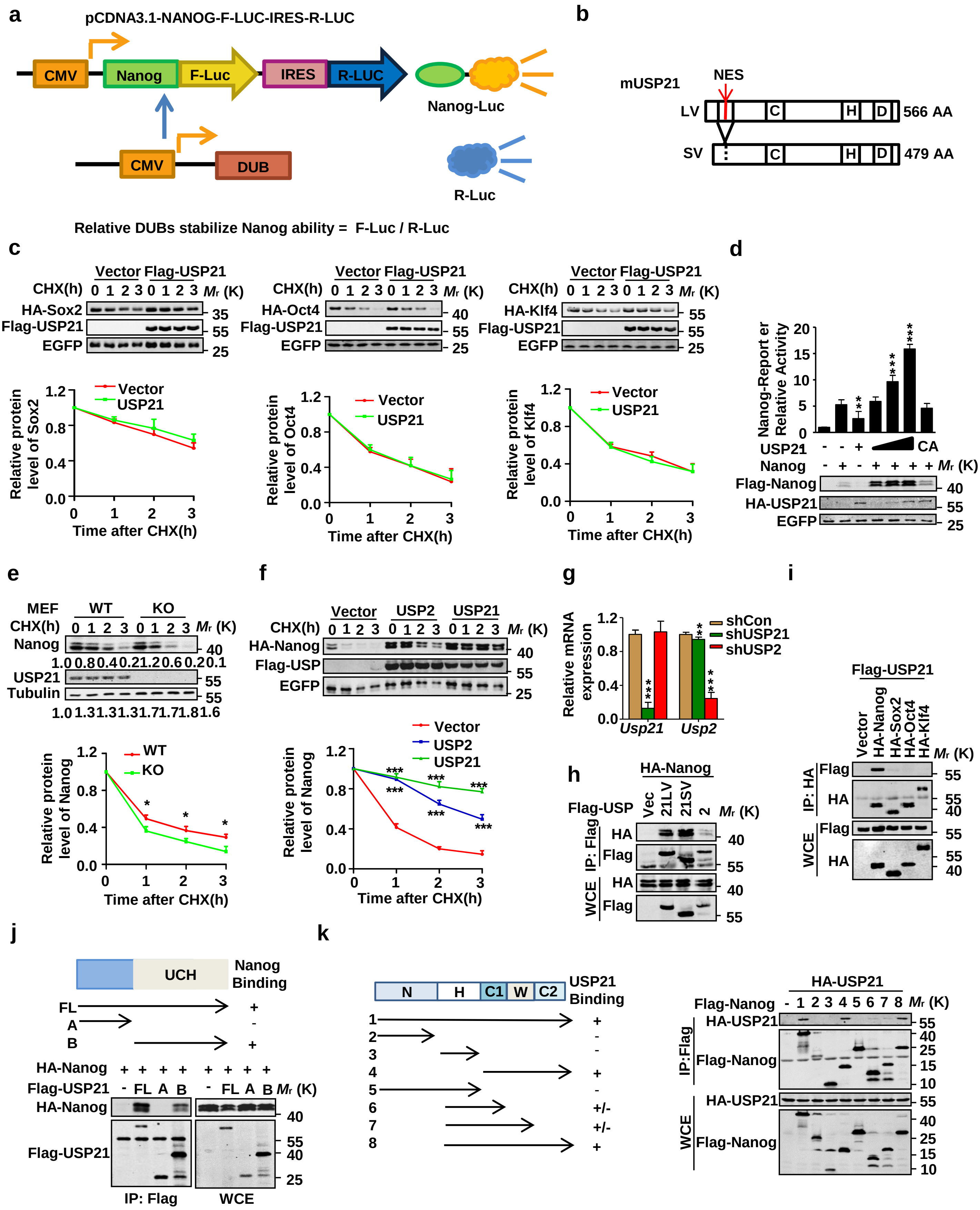
a
pCDNA3.1-NANOG-F-LUC-IRES-R-LUC
CMV
Nanog
F-Luc
IRES
R-LUC
Nanog-Luc
CMV
DUB
R-Luc
Relative DUBs stabilize Nanog ability = F-Luc / R-Luc
b
mUSP21
NES
LV
C
H
D
566 AA
SV
479 AA
c
Vector Flag-USP21
CHX(h) 0 1 2 3 0 1 2 3 Mr (K)
HA-Sox2
Flag-USP21
EGFP
HA-Oct4
HA-Klf4
Relative protein level of Sox2
Relative protein level of Oct4
Relative protein level of Klf4
Vector
USP21
Time after CHX(h)
d
Nanog-Report er Relative Activity
USP21
Nanog
CA
Flag-Nanog
HA-USP21
EGFP
e
MEF
WT
KO
Nanog
USP21
Tubulin
1.0 0.8 0.4 0.2 1.2 0.6 0.2 0.1
1.0 1.3 1.3 1.3 1.7 1.7 1.8 1.6
Relative protein level of Nanog
Time after CHX(h)
f
Vector
USP2
USP21
HA-Nanog
Flag-USP
EGFP
g
Relative mRNA expression
shCon
shUSP21
shUSP2
Usp21
Usp2
h
HA-Nanog
Flag-USP
Vec
21LV
21SV
2
IP: Flag
WCE
HA
Flag
i
Flag-USP21
Vector
HA-Nanog
HA-Sox2
HA-Oct4
HA-Klf4
IP: HA
WCE
j
UCH
Nanog Binding
FL
A
B
HA-Nanog
Flag-USP21
IP: Flag
WCE
k
N
H
C1
W
C2
USP21 Binding
+/-
HA-USP21
Flag-Nanog
IP:Flag
WCE

Supplementary Figure 1. USP21 directly stabilizes and interacts with Nanog. (a). A schematic represents screening model for Nanog deubiquitinase using dual luciferase reporter system. **(b).** Schemes of USP21LV and USP21SV. The alternatively spliced sequence in USP21SV is indicated with dashed line. **(c).** The half-life of stem cell transcriptional factors (Sox2, Oct4, Klf4). Statistical analysis was presented in the lower panel. Data are means±s.d. (n=3). **(d).** Nanog was co-transfected with USP21 WT or USP21 C221A together with Nanog-luciferase reporter. The firefly luciferase activity was measured and normalized to the renilla luciferase activity in the same sample and then normalized to vector control. Data are means±s.d. (n=3). **$P<0.05$, ***$P<0.001$ vs USP21(-) and Nanog(+) condition (Student's *t*-test). **(e).** WT and USP21 knockout MEFs were infected with virus carrying Flag-Nanog and then treated with CHX (10 μg ml^{-1}) for indicated time intervals, protein levels of Nanog and USP21 were analyzed by western blotting**.** Data are means±s.d. (n=3), *$P< 0.05$ vs WT (Student's *t*-test). **(f).** The half-life of Nanog upon overexpression of USP21 and USP2, respectively. Statistical analysis of Nanog was presented in the lower panel. Data are means±s.d. (n=3), ***$P< 0.001$ vs Vector (Student's *t*-test). **(g)**. The relative knockdown efficiency of USP21 and USP2 were detected by Real-time PCR. Data are means±s.d. (n=3), **$P< 0.01$,***$P< 0.001$ vs shCon (Student's *t*-test). **(h)**. Flag-USP2 and Flag-USP21 was coexpressed with HA-Nanog in HEK293T cells. The associated Nanog and USP2 or USP21 were analyzed by Western blotting. **(i).** The co-IP assay in HEK293T cells revealed that HA-Nanog could interact with USP21 instead of Sox2 or Oct4 or Klf4. **(j)**. Schematic diagram of USP21 (top panel). The domain of USP21 involved in its interaction with Nanog. **(k)**. Schematic diagram showing the structure of Nanog and its deletion constructs (left). N, interference domain; H, DNA-binding homeodomain; C (C1WC2), activation domain. The domain of Nanog involved in its interaction with USP21.

.

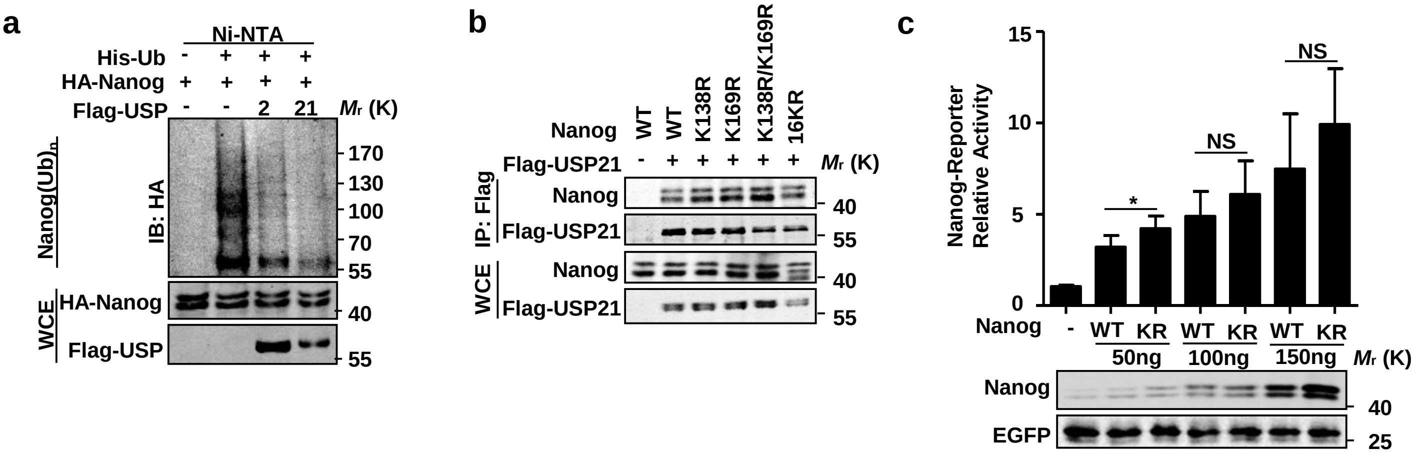

Supplementary Figure 2. USP21 deubiquitinates Nanog. (a). HA-Nanog and His-ubiquitin were coexpressed with USP2 or USP21 in HEK293T cells. After MG132 (10 μM) treatment for 6 hrs, the ubiquitinated proteins were pulled down under denaturing conditions using Ni-NTA agarose beads and the ubiquitination of Nanog was detected by western blotting using an anti-HA antibody. **(b).** WT or lysine-mutated Nanog and Flag-USP21 were coexpressed in HEK293T cells. After treatment with MG132 (10 μM) for 6 hrs, USP21 was immunoprecipitated with an anti-Flag antibody, and the associated Nanog and USP21 were analyzed by western blotting using either an anti-Nanog or Flag antibody. **(c).** WT or lysine-mutated Nanog was co-transfected with Nanog activity luciferase reporter gene into HEK293T cells for 24 hrs. The firefly luciferase activity was measured and normalized to the renilla luciferase activity in the same sample and then normalized to vector control. Data are means ± s.d. (n=3). *P<0.05 (Student's t-test) .

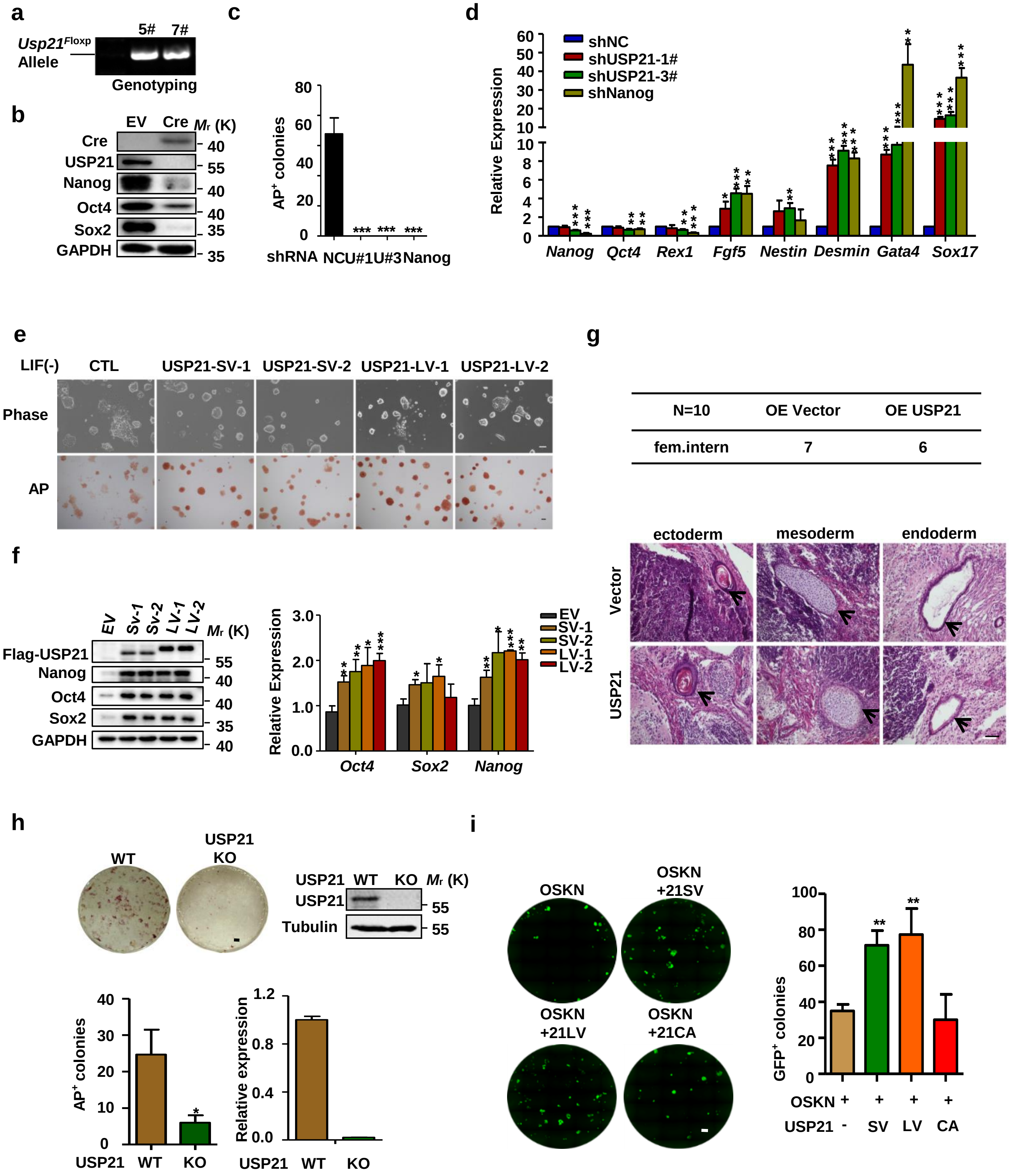

a
5#
7#
Usp21 Floxp Allele
Genotyping
b
EV
Cre
Mr (K)
Cre
USP21
Nanog
Oct4
Sox2
GAPDH
40
55
40
40
35
35
c
AP+ colonies
shRNA
NC
U#1
U#3
Nanog
***
d
Relative Expression
shNC
shUSP21-1#
shUSP21-3#
shNanog
Nanog
Oct4
Rex1
Fgf5
Nestin
Desmin
Gata4
Sox17
e
LIF(-)
CTL
USP21-SV-1
USP21-SV-2
USP21-LV-1
USP21-LV-2
Phase
AP
f
EV
SV-1
SV-2
LV-1
LV-2
Mr (K)
Flag-USP21
Nanog
Oct4
Sox2
GAPDH
55
40
40
35
40
Relative Expression
Oct4
Sox2
Nanog
g
N=10
OE Vector
OE USP21
fem.intern
7
6
ectoderm
mesoderm
endoderm
Vector
USP21
h
WT
USP21 KO
USP21
WT
KO
Mr (K)
USP21
Tubulin
55
55
AP+ colonies
Relative expression
USP21
WT
KO
i
OSKN
OSKN +21SV
OSKN +21LV
OSKN +21CA
GFP+ colonies
OSKN
USP21
SV
LV
CA

Supplementary Figure 3. USP21 is required for mESC self-renewal. (a). PCR was used to detect *Usp21*$^{Loxp/Loxp}$ ESCs. **(b).** Western blotting was used to detect Cre-induced loxp recombination in *Usp*21$^{Loxp/Loxp}$ ESCs infected with Cre lentivirus. The anti-USP21 antibody is from abgent. **(c).** Statistic of analysis of Figure 3B. Data are means$\pm$s.d. (n=3). ****P*< 0.001 vs shNC (Student's *t*-test)**. (d)**. Real-time PCR analysis of self-renewal and lineage markers upon E14 cells infected with virus carrying GFP together with control shRNA or shRNA targeting USP21 or Nanog. Ectoderm (ECTO), endoderm (ENDO), mesoderm (MESO). Data are means$\pm$s.d. (n=3). ***P*<0.01, ****P*<0.001 vs shNC (Student's *t*-test). **(e).** Morphology and AP staining of E14 cells stably expressing Flag-USP21SV/LV cultured in LIF-free condition for 3 days. Scale bar, 100 µm. **(f)**. The protein levels of USP21, Nanog, Sox2 and Oct4 in E14 cells stably expressing Flag-USP21 were analyzed by western blotting. Real-time PCR analysis of self-renewal markers in E14 cells stably expressing Flag-USP21. Data are means$\pm$s.d. (n=3). **P*<0.05, ***P*<0.01, ****P*<0.001 vs EV (Student's *t*-test). **(g)**. Teratoma formation from E14 cells infected with vector or USP21. Scale bar, 100 µm. **(h)**. AP intensity and colonies assessed at stages (day 12) of iPS induction in USP21WT and knockout MEF cells. Mean values$\pm$s.d. of a representative experiment measured in triplicate are shown. Scale bar, 1 mm. **P*<0.05 vs WT (Student's *t*-test). USP21 knockout MEF cells were tested by Real-time PCR and immunoblotting analysis. The anti-USP21 antibody is from Abgent. **(i)**. GFP^+ colonies assessed at stages (day 16) of iPSC induction in Oct4 GFP reporter MEF cell. Mean values$\pm$s.d. of a representative experiment measured in triplicate are shown. Scale bar, 1 mm. ***P*<0.01 vs OSKN condition (Student's *t*-test).

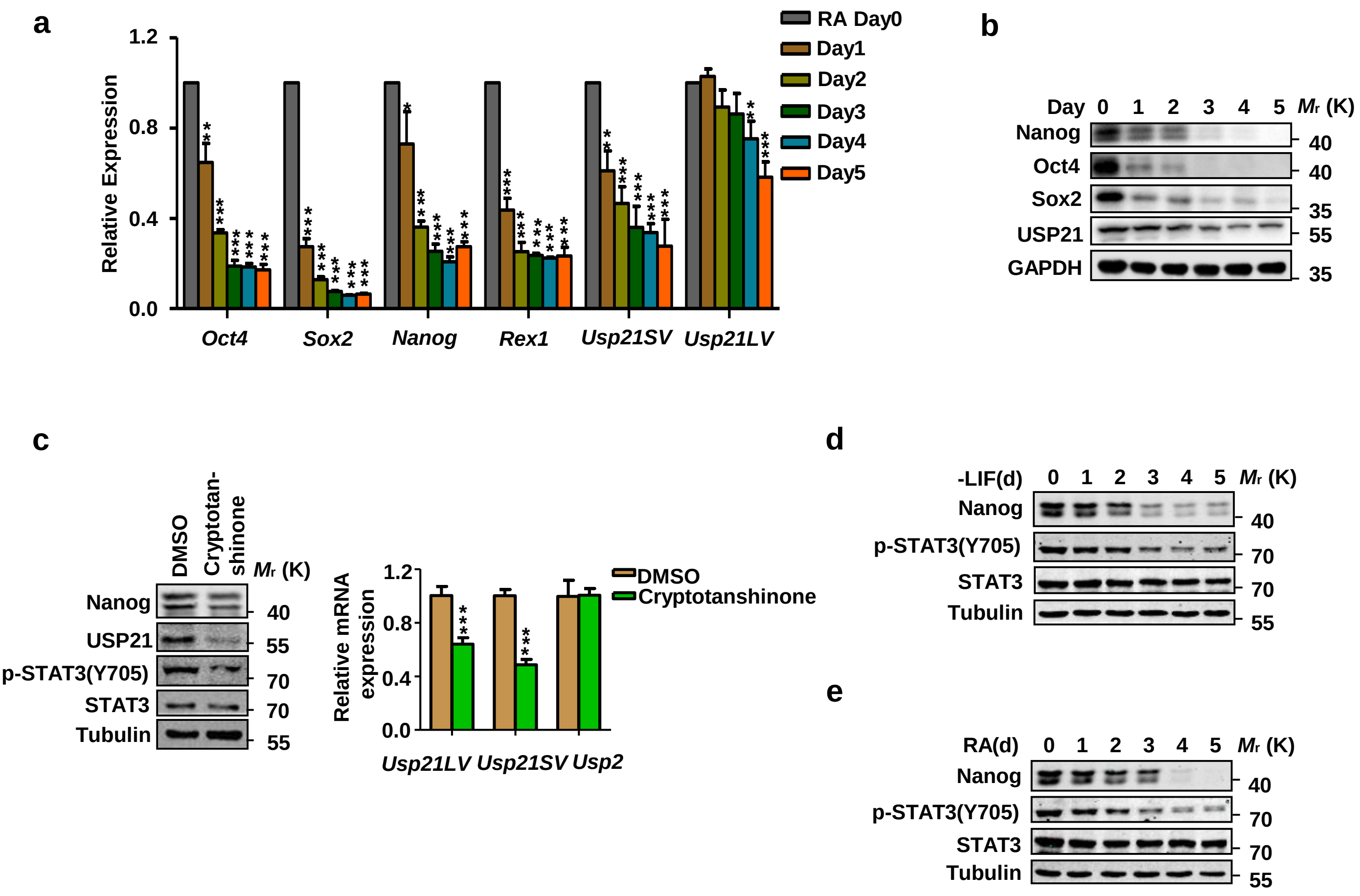

Supplementary Figure 4. USP21 is a transcriptional target of LIF/STAT3. (a and b). Real-time PCR (a) and immunoblot (b) analyses of pluripotency markers and USP21 in E14 cells cultured in RA containing medium for 5 days. Data are means±s.d. (n=3). *P<0.05, **P<0.01, ***P<0.001 vs Day0 (Student's *t*-test). USP21 was detected by an anti-USP21 from Abgent. **(c)**. Cryptotanshinone (10 μM) inhibited the expression of USP21. The expression of USP21 was detected by western blot and real-time PCR after treated with cryptotanshinone for 8 hrs. Data are means±s.d. (n=3). ***P<0.001 vs DMSO (Student's *t*-test). Anti-USP21 antibody is from abgent. **(d and e)**. The protein level of p-STAT3 decreased significantly by LIF withdraw (d) and RA treatment (e) in E14 cells.

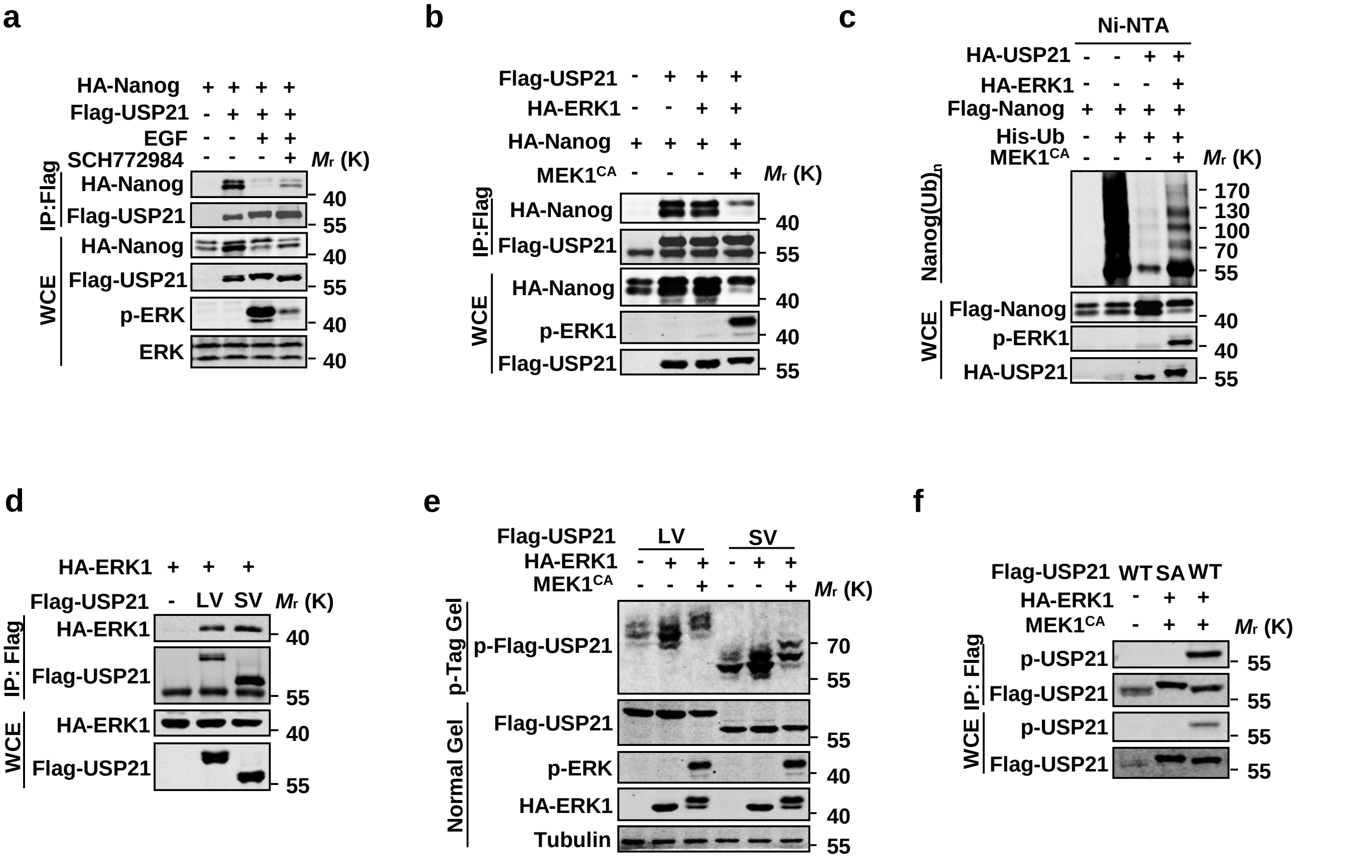

Supplementary Fig 5. USP21 is phosphorylated by ERK1. (a). Transfected HEK293T cells were treated with EGF (100 ng ml^{-1}) for 15 min and SCH772984 (10 μM) for 4 hrs. USP21 were immunoprecipitated with an anti-Flag antibody, and the associated Nanog was analyzed by western blotting using HA antibody. **(b).** Phosphorylation blocked the interaction between USP21 and Nanog. **(c).** Phosphorylation blocks USP21 mediated Nanog deubiquitination. The transfected HEK293T cells were treated with MG132 (10μM) for 6 hrs, the ubiquitinated proteins were pulled down under denaturing and analyzed by western. **(d).** Binding of ERK1 to USP21. **(e).** HEK293T cells were transfected with indicated combination of USP21, ERK1 and MEK1CA plasmids. Phos-Tag SDS-PAGE was applied to detect the band shift of USP21 caused by phosphorylation. Flag-USP21 LV/SV and HA-ERK1 were determined by regular SDS-PAGE with the anti-Flag or anti-HA antibodies, respectively. **(f).** The specificity of antibody against the phosphorylaed USP21 at S539.

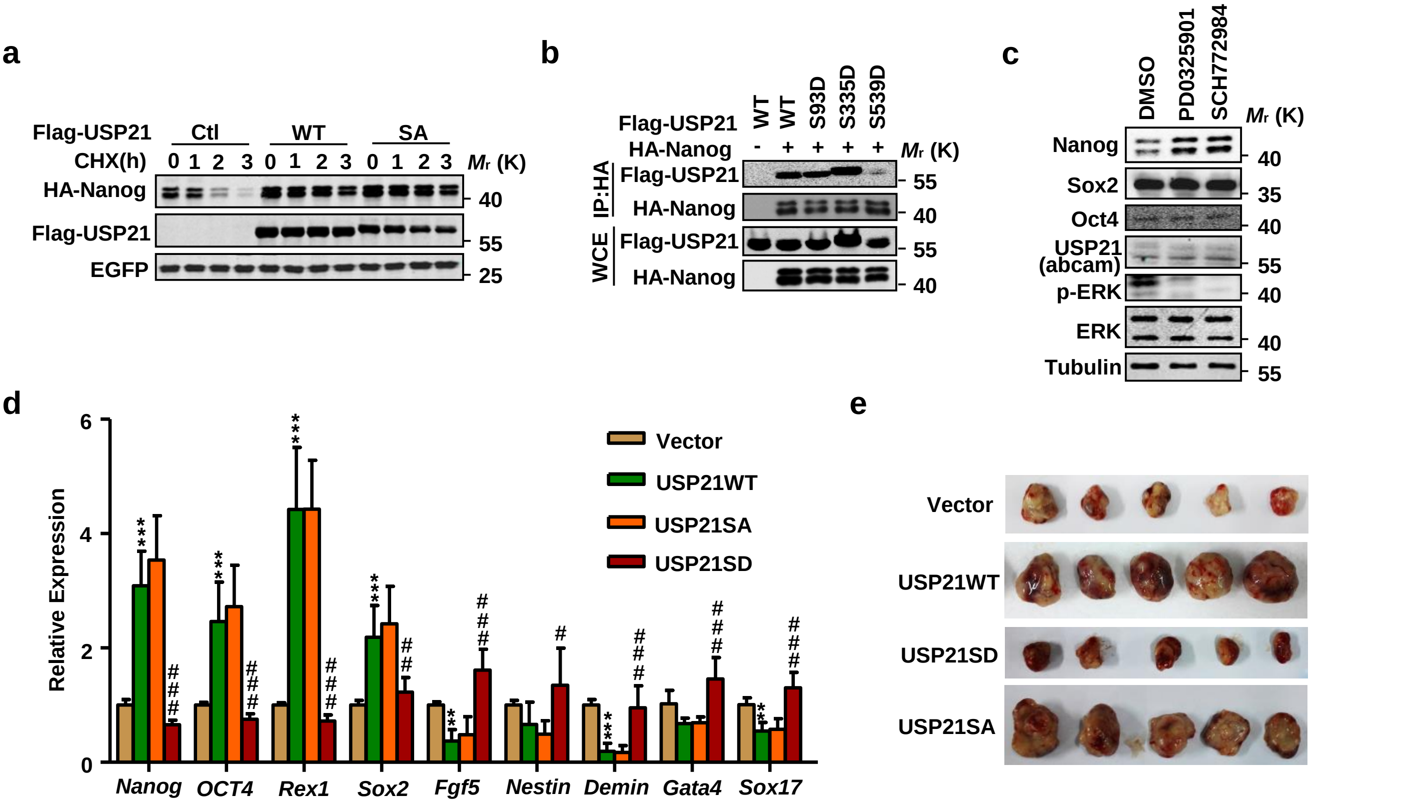

Supplementary Figure 6. Phosphorylation of USP21 at 539 blocks its effect on Nanog. (a). The effect of USP21 WT and S539A on the stability of HA-Nanog HEK293T cells. **(b).** S539D but not S93D and S335D blocked the binding of to Nanog. **(c).** Inhibition of ERK stabilized Nanog. E14 cells were cultured with LIF-free condition with the MEK inhibitor PD0325901 (1 μM) or ERK1/2 inhibitor SCH772984 (10 μM). Media were changed every 24 hrs over 4 days. The anti-USP21 antibody is from Abcam. **(d)**. The effect of USP21 and its mutants on the gene expression in E14 cells. E14 cells stably expressing USP21 (WT, S539A, S539D) were cultured in N2B27 medium overnight and treated with mFGF4 (25 ng ml^{-1}) for 12 hrs. Data are means±s.d. (n=3). **P<0.01, ***P<0.001 vs Vector (two-way ANOVA test); #P< 0.05, ##P< 0.01, ###P< 0.001 vs USP21 WT (two-way ANOVA test). **(e).** Tumor images were taken 5 weeks after inoculation.

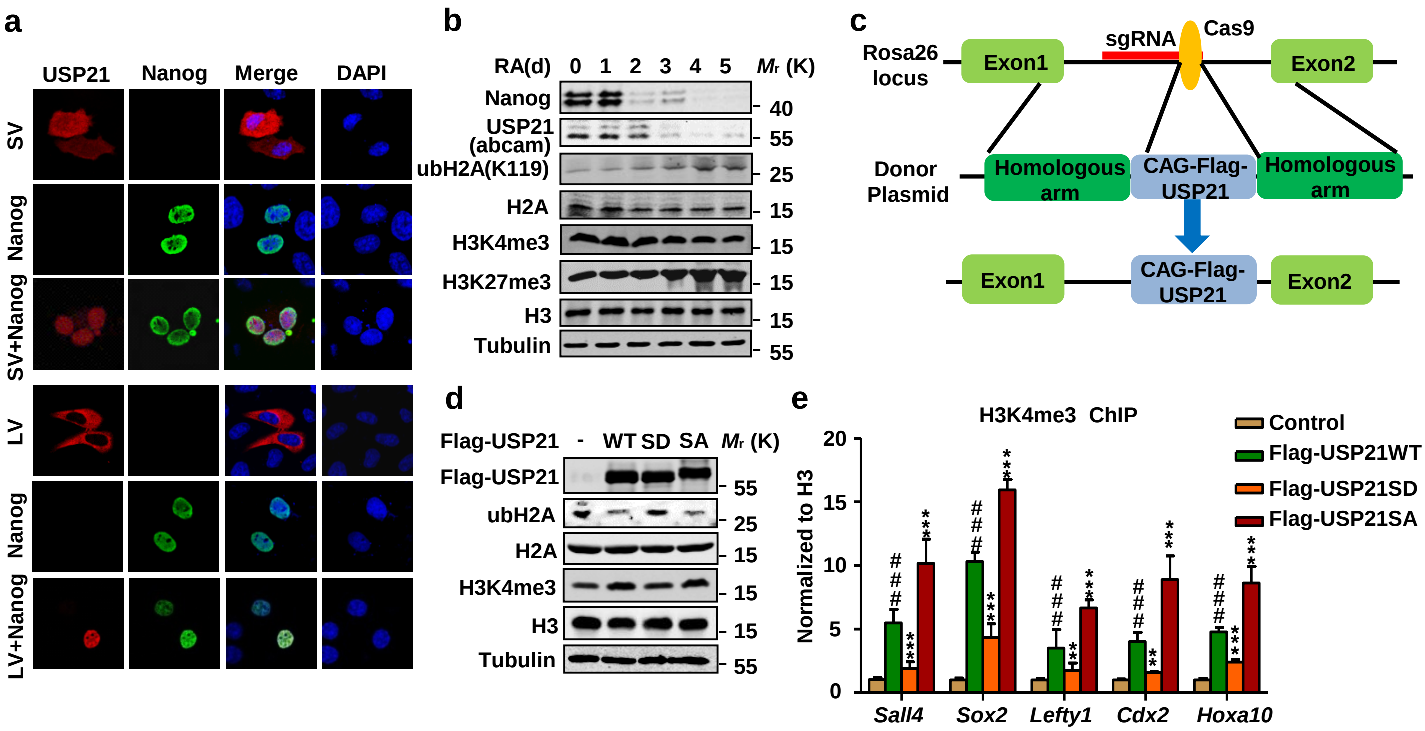

Supplementary Figure 7. USP21 is recruited to gene promoters and modulates H2A ubiquitination. (a). USP21 was re-localized to the nucleus by coexpressed Nanog. HA-Nanog was coexpressed with Flag-USP21LV/SV in HeLa cells. The subcellular location of Nanog (green) and USP21 (red) was examined by immunofluorescent staining (nuclei were stained with DAPI; blue). **(b).** The effect of RA treatment on histone ubiquitination and methylation in E14. The anti-USP21 antibody is from abcam. **(c).** Schematic overview of strategy to generate Flag-USP21 (WT, S539A, S539D) knock-in E14 stable cell line. **(d).** The effect of USP21 and its mutants on histone ubiquitination and methylation in E14 cells. **(e).** The effect of USP21 and its mutants on the level of H3K4me3 at Nanog regulated gene promoters in E14 cells. Data are means±s.d. (n=3). ***P*<0.01, ****P*<0.001 vs USP21WT (two-way ANOVA test); ###*P*< 0.001 vs Control (two-way ANOVA test).

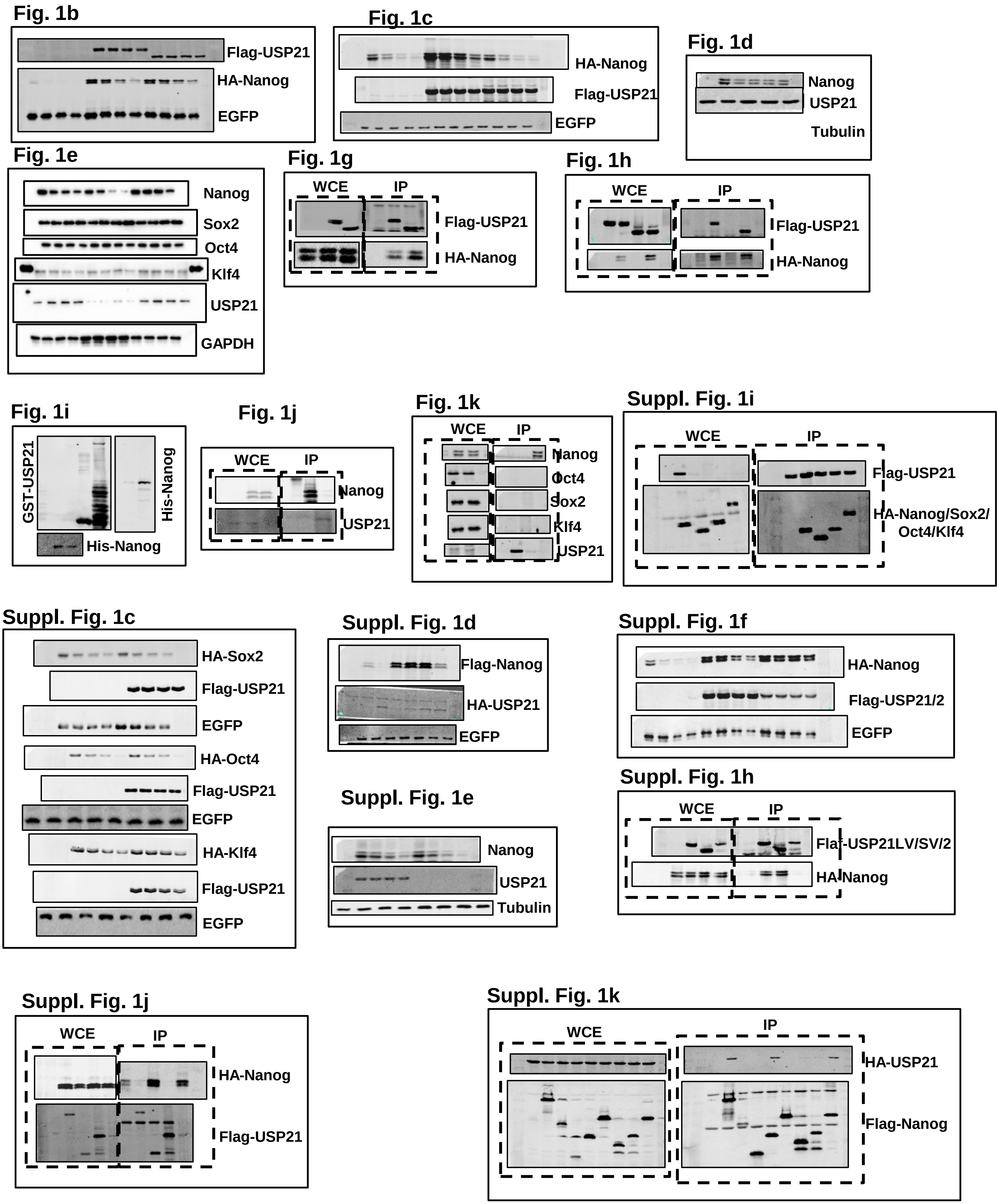

Supplementary Figure 8. Uncropped scans of blots. Uncropped scans of Figure 1 and Supplementary Figure 1.

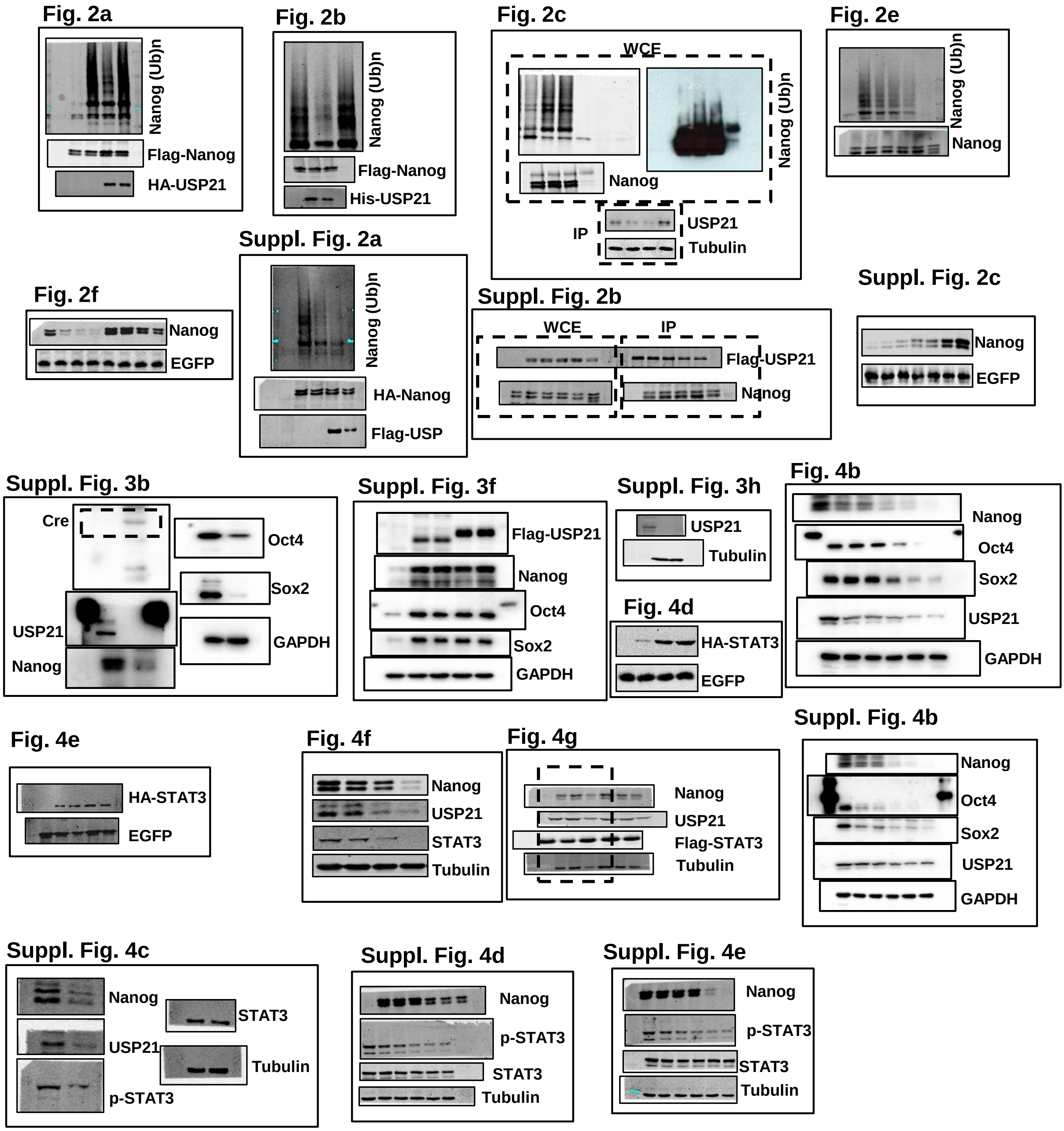

Supplementary Figure 9. Uncropped scans of blots. Uncropped scans of Figure 2-4 and Supplementary Figure 2-4.

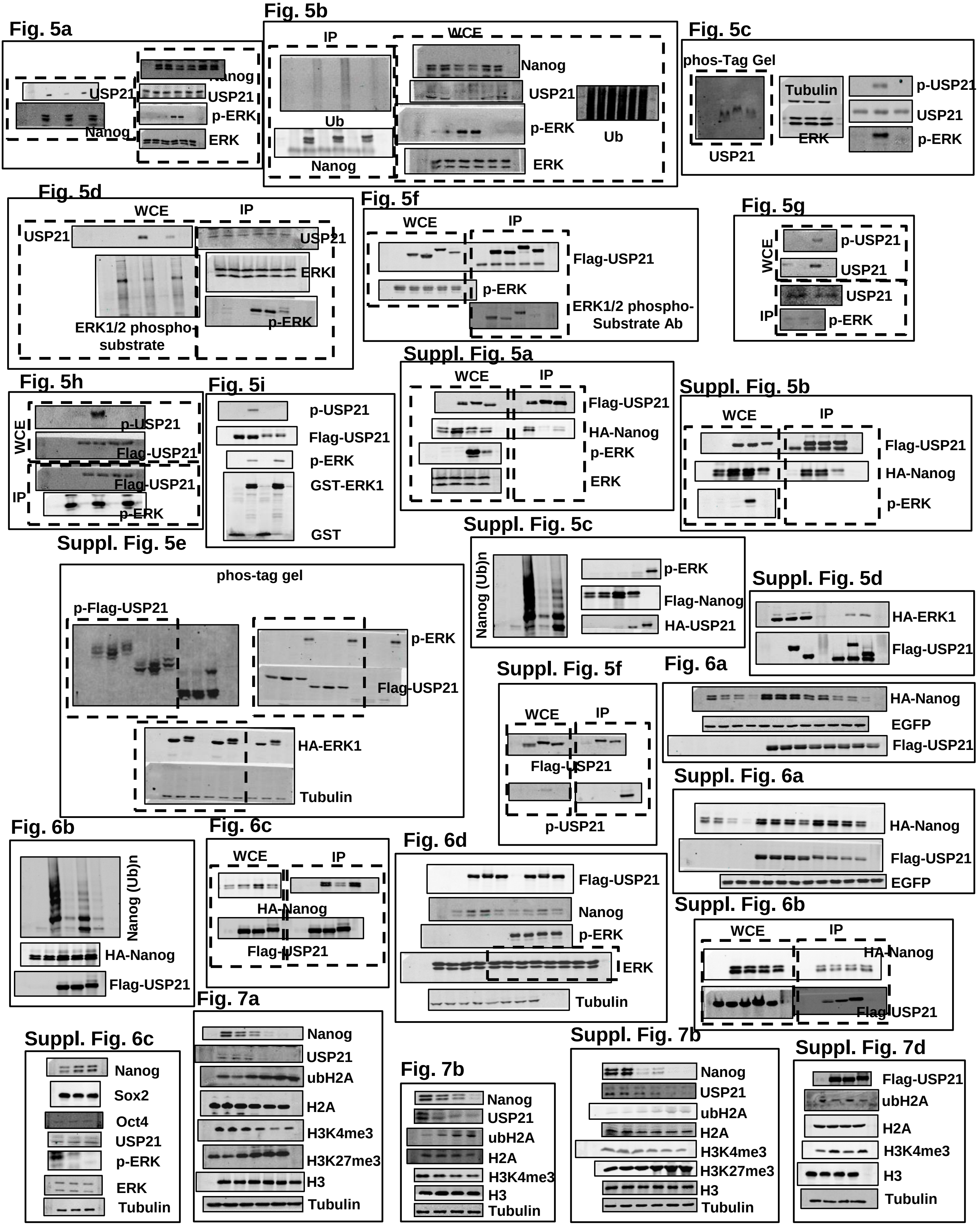

Supplementary Figure 10. Uncropped scans of blots. Uncropped scans of Figure 5-7 and Supplementary Figure 5-7.

Supplementary Table 1。Gene Expression Primers

Gene Expression primers		
**NAME**	**5' Fwd**	**3' Rev**
Nanog	CTCAAGTCCTGAGGCTGACA	TGAAACCTGTCCTTGAGTGC
Oct4	TAGGTGAGCCGTCTTTCCAC	GCTTAGCCAGGTTCGAGGAT
Rex1	GACGGATACCTAGAGTGCATCA	GAAGGGAACTCGCTTCCAGAA
Sox2	AGGGCTGGGAGAAAGAAGAG	CCGCGATTGTTGTGATTAGT
USP21SV	GATGAAAGGCTCAAGAAACTGGAG	TGCTGCTCAAACACTGTAGC
USP21LV	CATGGCTCCTTCCACATGAT	AGGGCACCAATCACATCTGC
Fgf5	GAAATATTTGCTGTGTCTCAGGG	TAAATTTGGCACTTGCATGG
Nestin	AGAAGACGGTGTGTCTGCTT	TAGAGGTGCAGCAGCTGCAG
Demin	TGACAACCTGATAGACGACC	TTAAGGAACGCGATCTCCTC
Gata4	TCCTACTCCAGCCCCTACC	GTAGTGTCCCGTCCCATCTC
Sox17	GAACAGTTGAGGGGCTACAC	GTTTAGGGTTTCTTAGATGC
USP2	CCATGGGAGGCCACTATACAGCCTA	CAAATAGGCGTCGCTGGT

Supplementay Table 2. Primers for ChIP-qPCR

ChIP-qPCR primers		
**NAME**	**5' Fwd**	**3' Rev**
GAPDH	AGTGCCAGCCTCGTCCCGTAGA CAAAATG	AAGTGGGCCCCGGC CTTCTCCAT
Sall4	TGGACAACCCAGAGCTGAAT	CAGGAGGGGCTGTCCTATTT
Sox2	CCAAGACTGGAGCTCACAATC	CAGGTGGAGCCTGAAAAGAAG
Lefty1	GTCCAGACAGGCTTTTGTGT	AGTCTGCGGAGGAATGGTA
Cdx2	GGACTCCGCGAGCCAA	CTCAGCCCACGGTGCTC
Hoxa10	CTGGCTCTTGAACCTGTACCCC	CAAGGGTGCTTCCAAATAGTC

SupplementaryTable 3. Nanog binding sites at genomic locations

Genesymbol	TSS	TTS	Refseq ID	chromsome
Sall4	91551142	91568061	NM_175303	chr2
Sox2	34548926	34551382	NM_011443	chr3
Lefty1	182865169	182868532	NM_010094	chr1
Cdx2	148112475	148118825	NM_007673	chr5
Hoxa10	52181195	52184938	NM_008263	chr6
